# Green synthesis, structural analysis and anticancer activity of dihydropyrimidinone derivatives†

**DOI:** 10.1039/d1ra03969e

**Published:** 2021-11-04

**Authors:** Jayanta Dowarah, Devanshi Patel, Brilliant N. Marak, Umesh Chand Singh Yadav, Pramod Kumar Shah, Pradeep Kumar Shukla, Ved Prakash Singh

**Affiliations:** Department of Chemistry, School of Physical Sciences, Mizoram University Aizawl-796004 Mizoram India vpsingh@mzu.edu.in; School of Life Sciences, Central University of Gujarat Gandhinagar Gujarat 382030 India; Special Centre for Molecular Medicine, Jawaharlal Nehru University New Delhi-110067 India; Department of Physics, Assam University Silchar-788011 India; Department of Industrial Chemistry, School of Physical Sciences, Mizoram University Aizawl-796004 Mizoram India

## Abstract

In this study, for the first time, we have used *Citrus macroptera* juice to synthesize dihydropyrimidine (DHPM) derivatives *via* the Biginelli reaction, which showed better yield, shorter reaction time, and did not require an organic solvent for the reaction. A series of DHPM derivatives were synthesized, and characterized, and structural analysis was achieved through SCXRD & Hirshfeld surface analysis. We observed that these synthesized dihydropyrimidine (DHPM) derivatives showed C–H⋯π, C–H⋯O, C–H⋯N, C–H⋯C, lone pair⋯π, π⋯π, *etc.* interactions. We also performed *in silico* studies for their inhibitory activities against human kinesin Eg5 enzyme, and the cytotoxic activity of the synthesized compounds was carried out against A549 lung adenocarcinoma cells. *In silico* analysis demonstrated that compounds with a chloro-group at the 3- or 4-position in the substituted ring of DHPM showed higher binding affinity for the human kinesin Eg5 enzyme (−7.9 kcal mol^−1^) than the standard drug monastrol (−7.8 kcal mol^−1^). Furthermore, *in vitro* cellular studies revealed that compounds with a chloro-group at the 3- or 4-position in the substituted ring of DHPM induced significant cell death in human A549 lung adenocarcinoma cells. This result indicates that a deactivating group (chlorine) at the 3- or 4-position in the substituted ring of DHPM might be a promising anticancer drug candidate for treating different types of cancers, particularly cancer of the lung.

## Abstract 2

Green synthesis, study and development of new potent dihydropyrimidinone analogues as anti-cancer drugs.

## Graphical abstract

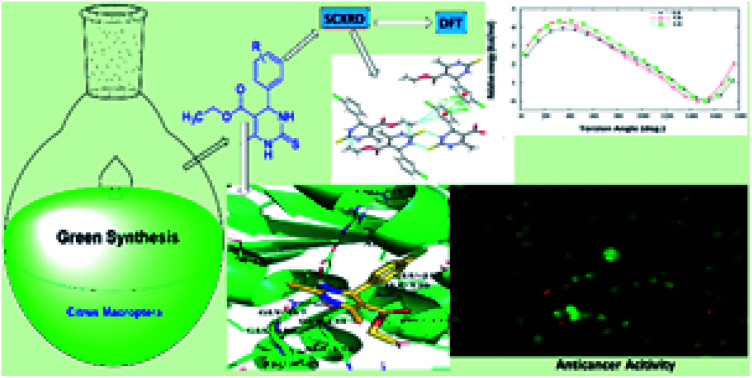

## 1. Introduction

Cancer accounts for the second leading cause of death worldwide despite many breakthroughs for cancer treatment. Almost 10.0 million cancer deaths occurred due to cancer in 2020 alone.^1^ According to the WHO, if this trend continues, this is expected to rise to 13.1 million by the end of 2030. Due to drug resistance and toxicity associated with its treatment, cancer remains a focus of immense interest.^2,3^ This warrants newer drug discovery and drug development efforts to mitigate these concerns in cancer therapy.

**Fig. 1:**
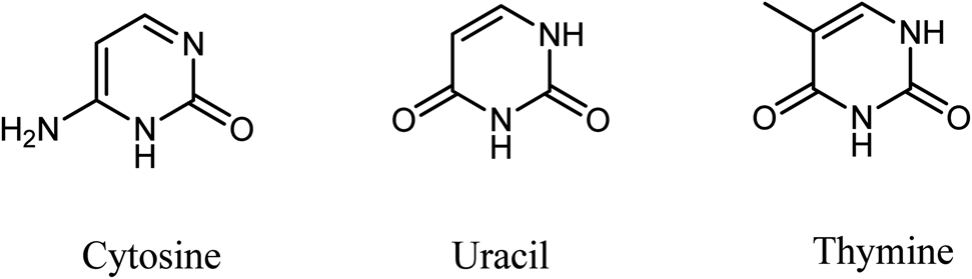
Pyrimidine derivative bases.

Heterocyclic molecules continue to be attractive targets for synthesis since they often exhibit vital biological properties. In particular, dihydropyrimidinones (DHPMs) are well known for their wide range of bioactivities. DHPMs have vital applications in the field of drug discovery. Three nucleic acid bases are pyrimidine derivatives, which comprise cytosine, found in DNA and RNA, uracil in RNA, and thymine in DNA (Fig. 1). Therefore, the synthesis and study of DHPM systems with methylene-linked ester makes it an exciting topic for synthetic organic chemistry.

**Fig. 2:**
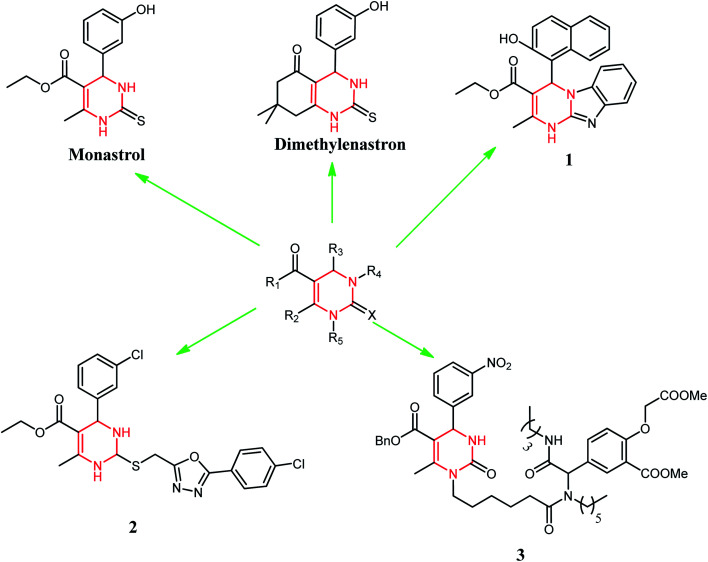
Dihydropyrimidinone (DHPM) scaffold as a potent anticancer drug.

DHPM classes of compounds are structural analogs of monastrol. These are important to medicinal chemistry because of many biological activities.^4^ These compounds also possess interesting biological activities such as antiviral,^5^ antitumor,^6^ and antibacterial,^7^ as well as calcium channel modulating activity.^8^ Monastrol is the protagonist of the DHPMs class. Several studies revealed that DHPMs inhibitory action on human kinesin Eg5 leads to a mitotic arrest resulting in apoptosis.^9^ This was the main action in apoptosis described for this class of compounds. However, some studies have shown other possible targets, such as centrin,^10^ calcium channels,^11^ and topoisomerase I^12^ for these molecules. Monastrol has inspired medicinal chemists to design new anticancer agents based on the DHPM scaffold or its modification with different substituents. Broad replacements (R_1_, R_2_, R_3_, R_4_, and X) were set on various ring places, meaning to get vital anticancer specialists, for example, dimethylenastron, compounds 1, 2, and 3 (Fig. 2). Thus, we have designed our synthetic compounds based on these structural attributes of monastrol.

The present study deals with the green synthesis of monastral analogues and studies weak interactions, including π⋯π interactions, C–H⋯π and lone pair⋯π interactions. Our first effort was to understand non-covalent interactions in the synthesized DHPM derivatives and the effect of substituents with different electronic environments on conformations of some selected synthesized molecules.

**Scheme 1:**
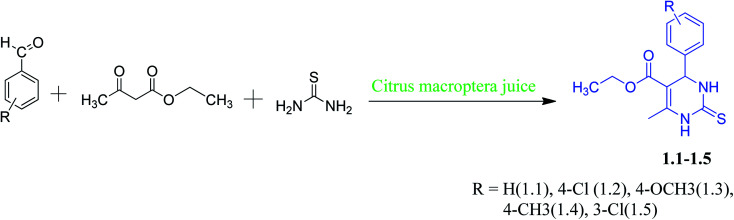
Green synthesis of DHPM derivaties (1.1–1.5).

The synthesis of DHPM and evaluation of their anticancer properties is of ongoing interest to our laboratory. Synthesis of DHPM derivatives by “solvent-free and catalyst-free” green methodology has been done by others.^13^ Pramanik *et al.* developed a safe, eco-friendly, and green method for synthesizing DHPM at room temperature using common fruit juice.^14^ Microwave-assisted green synthesis of DHPM was developed by Bhatewara *et al.* and studied their antimicrobial properties.^15^*Citrus macroptera* (a fruit from citrus family, usually found in India, Bangladesh, Malaysia and Melanesia) juice medium never have been used for Biginelli reaction using electron-rich/electron-deficient aromatic aldehydes, but similar kind of citrus fruit like orange and lime has been reported earlier for similar reactions.^14^ Here, we have reported a green synthesis for DHPM with electron-rich and electron-deficient aromatic aldehydes in *Citrus macroptera* juice medium [Scheme 1]. The non-covalent interactions of these compounds were studied by single-crystal X-ray diffractometers (SCXRD). Hirshfeld surface analyses were performed to study the structural property of molecules. DFT calculations were carried out to know how the substituents impact the geometry of different synthesized DHPM derivatives. *In silico* analysis studies the molecules binding affinity in the active site of target proteins (Eg5). Subsequently, we also carried out cytotoxic studies of these compounds in the lung adenocarcinoma (A549) cells.

## 2. Experimental design

^1^H NMR (300 MHz) and ^13^C (75 MHz) NMR spectra were recorded on JEOL AL300 FTNMR spectrometer using TMS as an internal reference, and chemical shift values are expressed in *δ*, ppm units. Melting points of all the compounds were recorded on the electrically heated instrument and were uncorrected. All the reactions were monitored by thin-layer chromatography (TLC) using an appropriate solvent system. The compound was purified from column chromatography (silica gel size 60–120 mesh) and flash chromatography (silica gel size 230–400 mesh) using the standard protocol.

### 2.1. Chemistry

Our main target was to perform green synthesis of DHPMs containing electron-rich and electron-deficient phenyl rings from *Citrus macroptera* juice and study the non-covalent interactions of these compounds through X-ray crystallographic study. The required *Citrus macroptera* juices were extracted directly from the naturally obtained *Citrus macroptera* fruit, and it was used straightaway for the reaction. DHPMs synthesis *via* Biginelli reaction with electron-rich and electron-deficient aromatic aldehydes in *Citrus macroptera* juice medium was done at room temperature (Scheme 1).

#### 2.1.1. General procedure for the synthesis of DHPM derivatives (1.1–1.5)

The equimolar quantities of ethyl acetoacetate (20 mmol, 2.6 g), thiourea (20 mmol, 1.52 g), aromatic aldehyde (20 mmol) were stirred in 4 mL of *Citrus macroptera juice* at room temperature for 12 h. Upon completion of the reaction, the solid product was precipitated out of the reaction medium. The crude solid product was filtered and recrystallized from hot ethanol to get the pure compounds (1.1–1.5) as pale-yellow solid.

##### 2.1.1.1. Ethyl-6-methyl-4-phenyl-2-thioxo-1,2,3,4-tetrahydropyrimidine-5-carboxylate (1.1)

Yield: 5.6 g, 83%, mp 205–207 °C. Solubility: ethanol, methanol

*δ*_H_ (300 MHz; CDCl_3_; Me_4_Si): 1.11–1.15 (3H, t, CH_3_, *J =* 7.2 Hz), 2.11–2.16 (3H, s, CH_3_), 4.15–4.18 (2H, q, CH_3_, *J =* 7.2 Hz), 5.29–5.37 (1H, s, CH), 7.22–7.35 (5H, s, Ph), 9.21–9.24 (1H, s, NH), 9.87–9.91(1H, s, NH). *δ*_C_ (75MHz; CDCl_3_): 14.2, 18.3, 58.6, 61.7, 104.2, 126.3, 126.9, 128.5, 143.3, 160.3, 167.2, 174.1. MS (*m*/*z*): 277.09 (M^+^). Element analysis: found: C, 60.64; H, 5.46; N, 10.33%. Calculated for C_14_H_16_N_2_O_2_S: C, 60.85; H, 5.84; N, 10.14%.

##### 2.1.1.2. Ethyl-4-(4-chlorophenyl)-6-methyl-2-thioxo-1,2,3,4-tetrahydropyrimidine-5-carbox ylate (1.2)

Yield: 4.4 g, 87%, mp 202–204 °C. Solubility: ethanol, methanol

*δ*_H_ (300 MHz; CDCl_3_; Me_4_Si): 1.12–1.16 (3H, t, CH_3_, *J =* 7.2 Hz), 2.31–2.39 (3H, s, CH_3_), 4.1–4.13 (2H, q, CH_2_, *J =* 7.2 Hz), 5.28–5.33 (1H, s, CH); 7.21–7.32 (4H, s, Ph), 9.27–9.34 (1H, s, NH), 9.88–9.95 (1H, s, NH). *δ*_C_ (75MHz; CDCl_3_): 14.2, 18.3, 58.6, 61.7, 104.2, 126.9, 128.5, 132.3, 143.3, 160.3, 167.2, 174.1. MS (*m*/*z*): 311.05 (M^+^). Element analysis: found: C, 54.21; H, 4.53; N, 9.12%. Calculated for C_14_H_15_ClN_2_O_2_S: C, 54.10; H, 4.86; N, 9.01%.

##### 2.1.1.3. Ethyl-4-(4-methoxyphenyl)-6-methyl-2-thioxo-1,2,3,4-tetrahydropyrimidine-5-carboxylate (1.3)

Yield: 4.1 g, 79%, mp 210–214 °C. Solubility: ethanol, methanol

*δ*_H_ (300 MHz; CDCl_3_; Me_4_Si): 1.11–1.23 (3H, t, CH_3_, *J =* 7.2 Hz), 2.32–2.39 (3H, s, CH_3_), 3.74–3.79 (3H, s, OCH_3_), 4.11–4.13 (2H, q, CH_2_, *J =* 8.4 Hz), 5.31–5.35 (1H, s, CH), 6.78–6.86 (2H, d, Ph, *J =* 8.4 Hz), 7.16–7.23 (2H, d, Ph, *J =* 8.4 Hz), 7.8–7.84 (1H, s, NH), 8.43–8.51 (1H, s, NH). *δ*_C_ (75MHz; CDCl_3_): 14.2, 18.3, 55.8, 58.3, 61.7, 104.2, 114.1, 125.7, 135.6, 158.6, 160.3, 167.2, 174.1. MS (*m*/*z*):307.10 (M^+^). Element analysis: found: C, 58.60; H, 5.84; N, 9.06%. Calculated for C_15_H_18_N_2_O_3_S: C, 58.80; H, 5.92; N, 9.14%.

##### 2.1.1.4. Ethyl-6-methyl-2-thioxo-4-(*p*-tolyl)-1,2,3,4-tetrahydropyrimidine-5-carboxylate (1.4)

Yield: 5.3 g, 85%, mp 209–2011 °C. Solubility: ethanol, methanol

*δ*_H_ (300 MHz; CDCl_3_; Me_4_Si): 1.12–1.23 (3H, t, CH_3_, *J =* 7.2 Hz), 2.26–2.32 (3H, s, CH_3_), 2.33–2.38 (3H, s, CH_3_), 4.1–4.13 (2H, q, CH_2_, *J =* 7.2 Hz), 5.28–5.33 (1H, s, CH), 7.08–7.12 (2H, d, Ph, *J =* 8.2 Hz) 7.14–7.23 (2H, d, Ph, *J =* 8.1 Hz), 8.81–8.86(1H, s, NH), 9.48–9.52 (1H, s, NH). *δ*_C_ (75 MHz; CDCl_3_): 14.2, 18.3, 21.3, 58.3, 61.7, 104.2, 126.8, 128.8, 136.4, 140.3, 160.3, 167.2, 174.1. MS (*m*/*z*): 291.11 (M^+^). Element analysis: found: C, 62.21; H, 6.11; N, 9.38%. Calculated for C_15_H_18_N_2_O_2_S: C, 62.04; H, 6.25; N, 9.65%.

##### 2.1.1.5. Ethyl-4-(3-chlorophenyl)-6-methyl-2-thioxo-1,2,3,4-tetrahydropyrimidine-5-carboxylate (1.5)

Yield: 4.5 g, 92%, mp 212–215 °C. Solubility: ethanol, methanol

*δ*_H_ (300 MHz; CDCl_3_; Me_4_Si): 1.13–1.21 (3H, t, CH_3_, *J =* 7.2 Hz), 2.24–2.37 (3H, s, CH_3_), 4.1–4.13 (2H, q, CH_2_, *J =* 7.2 Hz), 5.27–5.31 (1H, s, CH), 7.18–7.31 (4H, m, Ph), 9.37–9.44 (1H, s, NH), 9.98–10.03 (1H, s, NH). *δ*_C_ (75 MHz; CDCl_3_): 14.2, 18.3, 57.8, 61.7, 104.2, 125.0, 126.7, 126.8, 129.9, 134.1, 144.7, 160.3, 167.2, 174.1. MS (*m*/*z*): 311.05 (M^+^). Element analysis: found: C, 54.04; H, 4.45; N, 9.21%. Calculated for C_14_H_15_ClN_2_O_2_S: C, 54.10; H, 4.86; N, 9.01%.

### 2.2. X-ray crystallographic study

All crystal data has been used to solve and refine the crystal structure by SHELXL-97 ^16^ in the program suite WinGX (version 1.80.05).^17^ The molecular graphics were developed by using ORTEP-3 ^18^ software. For 3D structure visualization and packing diagrams, Mercury 3.3 software has been used throughout the experiment. PARST-95 and PLATON^19^ were used for geometrical calculations. Hydrogen bonds were represented by broken light green lines in the packing diagram. Carbon atoms were represented by grey color, hydrogen atoms white, oxygen atoms red, nitrogen atoms light blue, sulphur atoms yellow, chlorine atoms blue, *etc.*, in the packing diagram. Single-crystal X-ray data for compounds 1.3, 1.4, and 1.5 were collected with an Oxford Diffraction Xcalibur CCD diffractometer.

### 2.3. Hirshfeld surface analysis

Hirshfeld surface analysis^20^ is used to visualize intermolecular interactions in crystals using 3D molecular surface contours and 2D fingerprint plots. The Hirshfeld surface and associated 2D fingerprint plots for the compounds were calculated using Crystal Explorer 17. Differing van der Waals radii determined the distance between the points on the surface to a nucleus (atom) inside (di) and outside (de) the mean surfaces. The Hirshfeld surface, fingerprint plots, and interaction energies of compounds have been calculated using Crystal Explorer 17.^21^ The C–H bond lengths converted to normalized values. This is based on neutron diffraction results.^22^ The interaction energies of compounds have been calculated using the CE-B3LYP/6-31G(d,p) functional/basis set combination. Superposition energy (BSSE) was corrected for the basis set using the counterpoise (CP) method.^23^

### 2.4. Density functional theory (DFT) calculations

Prior to investigating the effect of substituents on the synthesized DHPM derivatives on their non-covalent interactions and anticancer drug property, it is of importance to understand how the substituents impact the geometry of different synthesized DHPM derivatives (1.3, 1.4 and 1.5). We, therefore, carried out a series of density functional theory (DFT) calculations with the hybrid *meta*-GGA functional M06-2X^24^ and Pople's basis set 6-31+G(d,p), as implemented in Gaussian 09 software suite,^25^ to evaluate the stability of different possible conformers formed due to twisting of the phenyl rings with respect to the non-aromatic dihydropyrimidinone ring in the synthesized DHPM derivatives. The M06-2X functional developed by Truhlar's group is reported to be a reliable functional for the study of relative stability of conformers, noncovalent interactions, rate constants and for thermochemistry of a wide variety of molecular systems.^26,27^

### 2.5. Biological activity of 1.1–1.5

RPMI-1640 cell culture medium was purchased from Hyclone (GE healthcare life sciences, Marlborough, USA), penicillin–streptomycin–amphotericin B (PSA) cocktail, 0.25% trypsin EDTA, and Fetal Bovine Serum (FBS) were bought from Gibco, Thermo-fisher Scientific (Waltham, MA, USA); [3-(4,5-dimethylthiazol-2-yl)-2,5-diphenyltetrazolium bromide] (MTT) was purchased from Sisco Research Laboratories Pvt. Ltd. (Maharashtra, India). Dimethyl sulfoxide (DMSO), acridine orange, trypan blue, ethidium bromide, and Hoechst 33342 were acquired from Sigma-Aldrich (St. Louis, MO, USA).

#### 2.5.1. Cell culture

Human adenocarcinoma A549 cell line was acquired from ATCC (Manassas, VA, USA) and grown in RPMI-1640 media complemented with 1% PSA and 10% FBS at 37 °C and 5% CO_2_. The medium was changed every alternate day, and cells were trypsinized after achieving the confluency.

#### 2.5.2. MTT assay

The cytotoxicity or viability study of compounds 1.1, 1.2, 1.3, 1.4, and 1.5 was done with A549 cells. Approximately 8000 cells were plated per well in a 96-well plate, and after attachment, cells were treated with different concentrations of compounds 1.1, 1.2, 1.3, 1.4, and 1.5. Media were removed from wells after 24 and 48 h, followed by incubation with 50 μl of MTT solution in PBS (5 mg mL^−1^) for 4 h. Formazan crystals were dissolved in DMSO (150 μl), and absorbance was taken at 570 nm in a multimode plate reader (Synergy H1 Hybrid Reader, Biotek; Winooski, VT, USA).

#### 2.5.3. Trypan blue assay

Trypan blue is an azo dye that stains dead cells with the compromised membrane in blue colour, and it does not stain live cells with the intact membrane, thus they appear clear. Nearly 60 000 cells were plated in 12-well plates, subjected to various doses of 1.2, 1.4, and 1.5 for 24 and 48 h. The cells were harvested and pelleted at 1200 rpm for 5 min. The cells were mixed in 200 μl 1× PBS after removing the supernatant, and 0.5% trypan blue dye was added. Cells were counted under a phase-contrast microscope (Zeiss, Germany).

#### 2.5.4. Acridine orange-ethidium bromide staining

To assess apoptosis and necrosis in A549 cells, nearly 60 000 cells were seeded in a 12-well plate followed by treatment with selected compounds 1.2 and 1.5 (200 μM) for 24 and 48 h time points. Afterward, cells were harvested and pelleted at 1200 rpm for 5 min. The cell mix was prepared in 50 μl of 1× PBS after removing the supernatant, and to 10 μl of cell aliquot 1 μl acridine orange (1 μg mL^−1^) and 1 μl ethidium bromide (1 μg mL^−1^) were added on a slide and mounted with a cover slip. The slides were subjected to fluorescence microscopy (Olympus, Japan), and photomicrographs were taken at 200×.

#### 2.5.5. Hoechst 33342 staining

Hoechst 33342 is a nucleic acid staining dye that is used to observe morphological changes in apoptotic cells. Hoechst 33342 assay was performed in a 12-well plate in which tissue cultured cover slips were placed. 60 000 cells per well were seeded and incubated at 37 °C in a humidified CO_2_ incubator and treated with a 200 μM concentration of 1.2 and 1.5 compounds. After treatment, media were removed, and cells were rinsed with PBS. 1 mL Hoechst 33342 stain (1 μg mL^−1^ in 1× PBS) was added to the wells and incubated in the dark for 15–20 min at 37 °C. Hoechst 33342 was removed, and cells were washed thrice with chilled 1× PBS. Cover slips were removed from the wells and placed upside down on the slide containing mounting media and subjected to fluorescent microscopy (Olympus, Japan), and photomicrographs were taken at 200×.

### 2.6. Molecular docking

Molecular docking analysis was performed using the AutoDock Vina.^28^ The crystal structures of the human kinesin Eg5 and survivin protein were retrieved from the RSCB protein data bank (PDB id: 3UIH and 1X88, respectively).^29^ The protein preparation was done in autodock tools^30^ by removing co-crystallized ligands, embedded water molecules, and cofactors. It was further processed by adding polar hydrogens and saved in the pdbqt format. The crystal structure of ligands in .cif format were converted to .pdb format in Mercury, which is then imported to autodock tools to add gasteiger charges and saved in pdbqt format. The grid parameters for docking with the kinesin Eg5 motor domain were determined based on the native ligand monastrol. However, for survivin protein, the grid parameters were assigned carefully to encompass the binding sites at the dimerization interface and allosteric site located near it. The parameter was set to 9 modes for analyzing binding affinity, and subsequently, crystal compounds **1.1–1.5** were subjected to molecular docking with prepared proteins. The docking results were validated by re-docking. Further, validation of parameters for Eg5 was done by repeating the docking of the crystal structure of monastrol, which was extracted from Eg5 protein, then superimposing with native monastrol in Eg5 protein. The docked monastrol presented an RMSD value of 0.210 Å concerning native monastrol. Pymol and Discovery studio visualizer was used to analyze the docking results.

## 3. Results and discussion

A series of DHPM derivatives (1.1–1.5) were synthesized using urea, ethyl acetoacetate with electron-rich and electron-deficient aromatic aldehydes through green catalyzed reaction given in Scheme 1. First-time *Citrus macroptera* juices were used as bio-catalyst for the synthesized DHPM derivatives, which showed better yield and shorter duration. This procedure is also an eco-friendly one, as it does not require any organic solvent for the reaction. Out of five compounds, three compounds (1.3–1.5) crystallized as single crystals. Cytotoxic activity of synthesized compounds was carried out against A549 lung adenocarcinoma cells to assess their anticancer activity.

### 3.1. X-ray crystallographic studies of 1.3–1.5

**Fig. 3:**
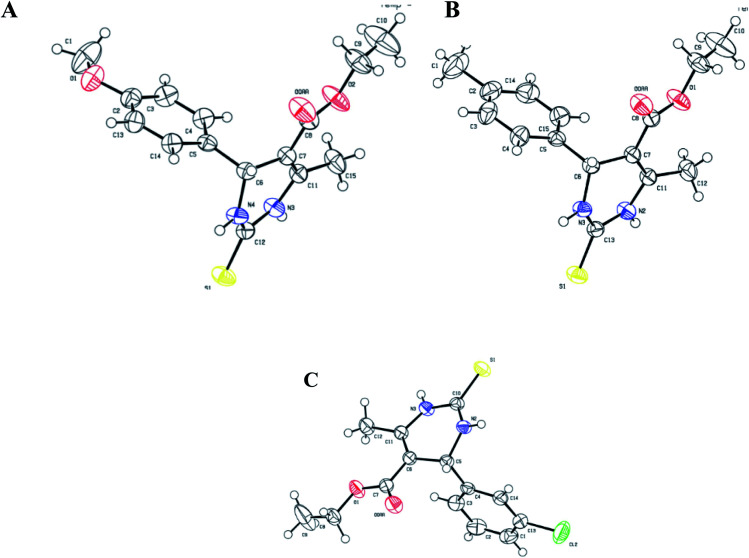
ORTEP diagram of ellipsoids at 50% probability level with the atomic numbering scheme for: (A) 1.3, (B) 1.4, and (C) 1.5.

All three prismatic transparent crystals (1.3, 1.4, and 1.5) were obtained using methanol solvent by slow evaporation, after 15 days, at room temperature. The ORTEP diagrams of 1.3, 1.4, and 1.5 are shown in Fig. 3. In all three crystal structures, the non-aromatic dihydropyrimidinone ring and phenyl ring occupied different planes. Compounds 1.4 and 1.5 crystallized in triclinic space group *P*1̄ with two independent molecules in the unit cell. Compound 1.3 crystallized in monoclinic space group *C*2/*c*, having eight molecules in the unit cell. Further details of crystal data and refinement are presented in Table 1.

**Table 1:** Crystal data and structure refinement for 1.3, 1.4 and 1.5

Crystal data	1.3	1.4	1.5
Identification code	1936021	1936022	1936023
Empirical formula	C_15_H_18_N_2_O_3_S	C_15_H_18_N_2_O_2_S	C_14_H_15_N_2_O_2_SCl
Formula weight	306.39	290.39	310.81
Temperature (K)	296 (2)	296 (2)	296 (2)
Crystal system	Monoclinic	Triclinic	Triclinic
Space group	*C*2/*c*	*P*1̄	*P*1̄
*a* (Å)	18.2332 (17)	7.3603 (10)	7.3066 (6)
*b* (Å)	7.3341 (6)	9.4648 (12)	10.4657 (8)
*c* (Å)	25.197 (2)	12.2076 (16)	10.6788 (9)
*α* (°)	90	74.216 (4)	107.568 (3)
*β* (°)	101.888 (4)	88.729 (4)	90.538 (3)
*γ* (°)	90	69.819 (4)	107.829 (2)
Volume (Å^3^)	3297.2 (5)	765.70 (18)	736.37 (10)
*Z*	8	2	2
*ρ* (g cm^−3^)	1.2343	1.2594	1.4016
*μ* (mm^−1^)	0.207	0.214	0.403
*F* (000)	1297.5879	308.3750	324.6689
Crystal size (mm^3^)	0.24 × 0.22 × 0.22	0.22 × 0.20 × 0.18	0.26 × 0.25 × 0.20
Radiation	Mo Kα (*λ* = 0.71073)	Mo Kα (*λ* = 0.71073)	Mo Kα (*λ* = 0.71073)
2*Θ* range for data collection (°)	2.28 to 28.37	2.96 to 2715	3.38 to 27.16
Reflections collected	39 480	18 180	14 725
Independent reflections	4117	3387	3263
Data/restraints/parameters	4117/0/201	3387/0/192	3263/0/191
Goodness-of-fit on *F*2	1.0714	1.0675	1.0760
Final *R* indexes [*I* ≥ 2*σ* (*I*)]	*R*1 = 0.0636, w*R*_2_ = 0.1885	*R*1 = 0.0423, w*R*_2_ = 0.1218	*R*1 = 0.0384, w*R*_2_ = 0.1104
Final *R* indexes [all data]	*R*1 = 0.0764, w*R*_2_ = 0.2033	*R*1 = 0.0487, w*R*_2_ = 0.1298	*R*1 = 0.0429, w*R*_2_ = 0.1156
Largest diff. peak/hole/e Å^−3^	0.57/−0.43	0.34/−0.38	0.28/−0.43

**Fig. 4:**
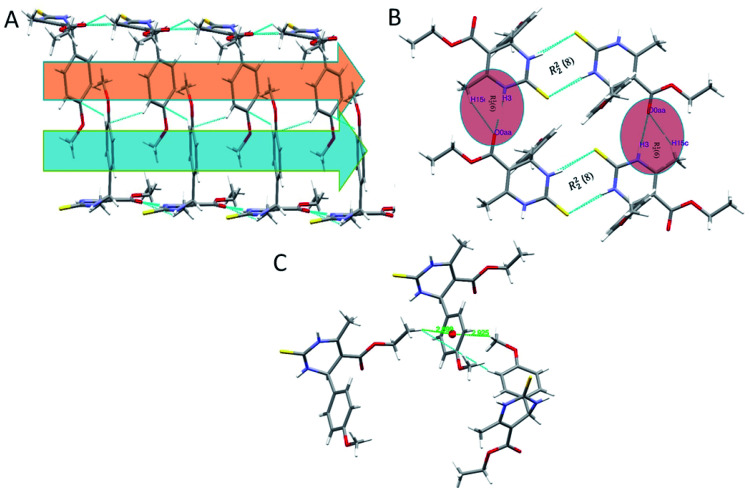
(A) Partial packing diagram of 1.3 showing C–H⋯π interactions with symmetry code 1.5 − *x*, −1/2 + *y*, 1.5 − *z* in layer A, layer B, (B) showing C–H⋯O, N–H⋯O, and N–H⋯S interactions forming R^2^_2_ (8) and R^1^_2_ (6) ring motifin 1.3, and (C) C–H⋯π interactions with symmetry code 1.5 − *x*, −1/2 + *y*, 1.5 − *z* and −1/2 + *x*, 1/2 + *y*, *z*.

In the crystal structure of compound 1.3, the crystal packing is stabilized by dimers through N–H⋯S hydrogen bonds in a R^2^_2_ (8) ring motif (Fig. 4). One more intermolecular hydrogen bonding of N–H⋯O & C–H⋯O results in the generation of a dimeric structure, which can be described as R^1^_2_ (6) graph-set notation in which thioamide & ester oxygen are involved (Fig. 4B). The hydrogen-bonding network for compound 1.3 and crystal packing are given in Fig. 4. Compound 1.3 exhibits a stacked arrangement of molecules. They exhibit a combination of parallel-displaced C–H⋯π, C–H⋯O, N–H⋯O, and N–H⋯S interactions (Table 2). D is the donor, and A is the acceptor in non-covalent interactions in Table 2. Both rings are arranged in an ABBA pattern in crystal packing. The acute angle between the plane of the non-aromatic ring and phenyl ring is 87.68°. The stacking distances for C–H⋯π were 2.669, 2.925, and 3.376 Å (Table 2 and Fig. 4C). The N–H⋯O and N–H⋯S bond distances for hydrogen bonding were 2.184, 2.463 Å, and angles on hydrogen atoms were 169.84°, 172.54°. Apart from these interactions, the C–H⋯O interactions also assist the interlayer connectivity and linear chain formation. The C–H⋯O bond distances for hydrogen bonding were 2.716, 2.770, 2.918 & 2.953 Å, and angles on hydrogen atoms were 132.54°, 137.19°, 119.22, and 118.79°, respectively (Table 2 & Fig. 4).

**Table 2:** Intermolecular and intramolecular interactions in 1.3

D–H⋯A	D–H (Å)	H⋯A (Å)	D⋯A (Å)	D–H⋯A (°)	Symmetry operation
**Compound** **1.3**					
C1–H1c⋯Oaa	0.959	2.770	3.534	137.19	1.5 − *x*, −1/2 + *y*, 1.5 − *z*
N4–H4⋯S1	0.862	2.463	3.316	169.84	1.5 − *x*, 1/2 − *y*, 1 − *z*
C3–H3a⋯O1	0.930	2.918	3.468	119.22	1.5 − *x*, −1/2 + *y*, 1.5 − *z*
C4–H4a⋯O1	0.930	2.953	3.497	118.79	1.5 − *x*, −1/2 + *y*, 1.5 − *z*
C15–H15c⋯O0aa	0.960	2.716	3.439	132.54	*x*, −1 + *y*, *z*
N3–H3⋯O0aa	0.804	2.184	2.983	172.54	*x*, −1 + *y*, *z*
C10–H10a⋯S1	0.961	3.251	4.001	137.89	−1/2 + *x*, 1/2 + *y*, *z*
C14–H14⋯S1	0.930	3.144	3.875	136.92	*x*, −1 + *y*, *z*
C6–H6⋯S1	0.980	3.182	4.095	155.77	*x*, −1 + *y*, *z*
C1–H1a⋯π (C2, C3, C4, C5, C13, C14)	0.960	3.627	3.684		1.5 − *x*, −1/2 + *y*, 1.5 − *z*
C1–H1b⋯π (C5, C4, C2, C3, C13, C14)	0.961	2.925	3.684		1.5 − *x*, −1/2 + *y*, 1.5 − *z*
C10–H10c⋯π (C5, C4, C2, C3, C13, C14)	0.959	2.699	3.641		−1/2 + *x*, 1/2 + *y*, *z*

**Intramolecular**					
C3–H3a⋯O1	0.930	2.644	2.443		
C13–H13a⋯O1	0.930	2.492	2.341		
C9–H9a⋯O0aa	0.970	2.588	2.675		
C9–H9b⋯O0aa	0.970	2.736	2.675		
C15–H15c⋯N3–H3	0.960	2.125	2.394		

**Compound** **1.4**					
C12–H12c⋯Oaa	0.960	2.713	3.403	129.33	−1 + *x*, *y*, *z*
C4–H4⋯S1	0.930	3.063	3.731	130.12	−1 + *x*, *y*, *z*
C6–H6⋯S1	0.980	3.268	4.146	150.03	−1 + *x*, *y*, *z*
N3–H3⋯S1	0.889	2.450	3.322	166.91	1 − *x*, −*y*, −*z*
N2–H2⋯O0aa	0.810	2.257	3.058	170.02	−1 + *x*, *y*, *z*
C12–H12a⋯Oaa	0.960	2.713	3.403	129.33	−1 + *x*, *y*, *z*

**Intramolecular**					
C15–H15⋯π (C6, C7, C11, C13, N2, N3)	0.931	2.846	3.067		
C12–H12a⋯O1	0.960	2.914	2.800		
C12–H12b⋯O1	0.960	2.317	2.800	110.40	
C6–H6⋯O0aa	0.980	2.474	2.812		
C9–H9a⋯O0aa	0.970	2.546	2.686		

**Compound**1.5					
C1–H1⋯O0aa	0.980	2.747	3.684	159.68	−1 + *x*, *y*, *z*
C12–H12a⋯O0aa	0.960	3.456	3.390	119.22	−1 + *x*, *y*, *z*
C12–H12c⋯O0aa	0.960	2.705	3.390	128.86	−1 + *x*, *y*, *z*
N3–H3⋯O0aa	0.805	2.186	2.981	189.86	−1 + *x*, *y*, *z*
C8–H8a⋯Cl2	0.970	2.952	3.767	142.39	−*x*, 1 −*y*, 1 − *z*
N2–H2⋯S1	0.829	2.584	3.401	188.89	1 − *x*, 1 − *y*, 2 − *z*
C5–H5⋯S1	0.980	3.213	4.126	155.57	−1 + *x*, *y*, *z*
C8–H8b⋯S1	0.970	3.098	4.024	160.25	1 − *x*, 2 − *y*, 2 − *z*
C9–H9a⋯S1	0.960	3.385	4.211	145.54	1 − *x*, 2 − *y*, 2 − *z*
C14–H14⋯S1	0.980	3.060	3.888	149.30	1 − *x*, 2 − *y*, 2 − *z*
C9–H9c⋯π (C1, C2, C3, C4, C13, C14)	0.959	3.180	4.128		*x*, −1 + *y*, *z*
C2–H2a⋯π (C2, C1, C13, C14, C4, C3)	0.930	3.611	3.874		−*x*, 1 − *y*, 1 − *z*
C1–H1⋯π (C1, C2, C3, C4, C13, C14)	0.930	3.480	3.369		−*x*, 1 − *y*, 1 − *z*
(C1, C2, C3, C4, C13, C14) π⋯π (C3, C4, C14, C13, C1, C2)		3.665			−*x*, 1 − *y*, 1 − *z*

**Intramolecular**					
C5–H5a⋯O0aa	0.980	2.449	2.786		
C8–H8a⋯O0aa	0.970	2.578	2.670		
C8–H8b⋯O0aa	0.970	2.732	2.670		
C12–H12a⋯O1	0.960	2.881	2.786		
C12–H12b⋯O1	0.960	2.309	2.786		

**Fig. 5:**
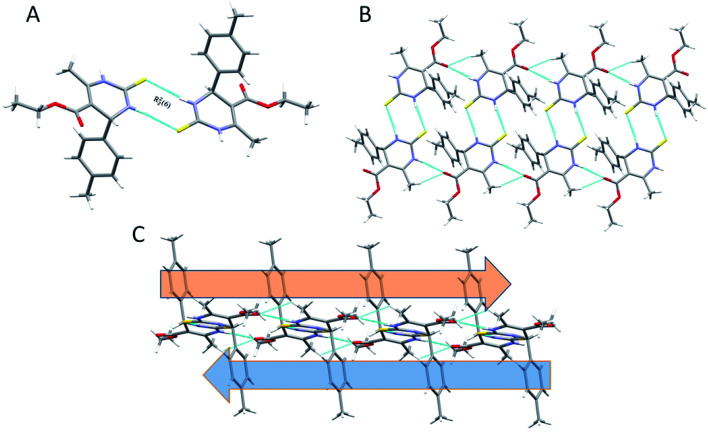
(A) Partial packing diagram of 1.4 forming R^2^_2_ (8) ring motif with symmetry code 1 − *x*, −*y*, −*z* (B) showing N–H⋯O, C–H⋯O & N–H⋯S interactions in 1.4 (C)partial packing diagram showing an alternate layer of the ring in the alternate pattern.

The overall structure of 1.4 exhibits a combination of C–H⋯O, N–H⋯O, and N–H⋯S interactions (Table 2). Phenyl rings are arranged in a hairbone pattern in crystal packing. The acute angle between the plane of the non-aromatic ring and phenyl ring is 80.95°. Dimers also stabilize the crystal packing of compound 1.4 through N–H⋯O, N–H⋯S, & C–H⋯O hydrogen bonds in R^1^_2_ (6), R^2^_2_ (8), and R^4^_4_ (20) ring motif, where thioamide & ester oxygen are involved (Fig. 5A and B). The hydrogen-bonding network for compound 1.4 and crystal packing are shown in Fig. 5. In addition to intermolecular C–H⋯O interactions compound, 1.4 also showed intra-molecular C–H⋯O interactions. The N–H⋯O and N–H⋯S bond distances for hydrogen bonding were 2.257, 2.450 Å, and angles on hydrogen atoms were 170.02°, 166.91°, respectively. The C–H⋯O bond distance and angle on hydrogen atom were 2.713 Å and 129.33°, respectively (Table 2 and Fig. 5). An intramolecular C–H⋯π interactions (H15⋯Cg = 2.846 Å) were also observed, which further stabilized interlayer connectivity of crystal structure.

**Fig. 6:**
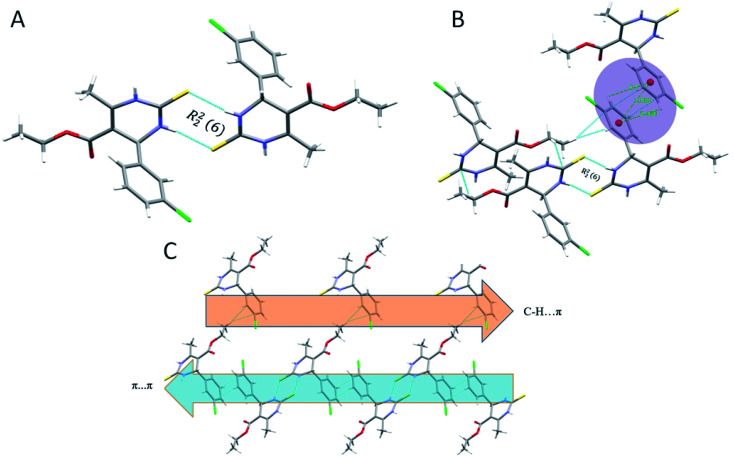
(A) Partial packing diagram of 1.5 forming R^2^_2_ (8) ring motif with symmetry code 1 − *x*, 1 − *y*, 2 − *z*, (B) π⋯π and C–H⋯π interactions in 1.5, and (C) partial packing diagram of 1.5 showing C–H⋯π interactions in layer A, and π⋯π stacking interactions in layer B.

Crystal structure of 1.5 exhibits a combination of parallel-displaced C–H⋯π, C–H⋯O, N–H⋯O, and N–H⋯S interactions (Table 2). Both rings are arranged in the AABB pattern in crystal packing. The acute angle (dihedral angle) between the non-aromatic ring and phenyl ring plane is 80.18°. Crystal 1.5 also stabilized like the rest two crystals by the dimers through N–H⋯S, N–H⋯O & C–H⋯O non-traditional hydrogen bonds resulting in the R^1^_2_ (6), R^2^_2_ (8), and R^4^_4_ (20) graph-set notation (Fig. 6). For crystal structure of 1.5, one π⋯π stacking interaction was also observed, and distance for π⋯π stacking interaction was 3.665 Å (Fig. 6B). The stacking distances for C–H⋯π were 3.180, 3.611, 3.480 Å (Table 2), which further assisted the interlayer connectivity and linear chain formation of crystal structure. The N–H⋯O and N–H⋯S bond distances for hydrogen bonding were 2.1876, 2.584 Å, and angles on hydrogen atoms were 138.86°, 168.69°, respectively (Table 2 and Fig. 6). The C–H⋯O bond distance for R^1^_2_ (6) ring motif is 2.705 Å and angles on hydrogen atoms was 128.86°, while the other C–H⋯O bond distance were 2.747, 3.456 Å and angle on hydrogen atom were 159.68 and 119.28°, respectively (Table 2).

### 3.2. Hirshfeld surface analysis of 1.3–1.5

**Fig. 7:**
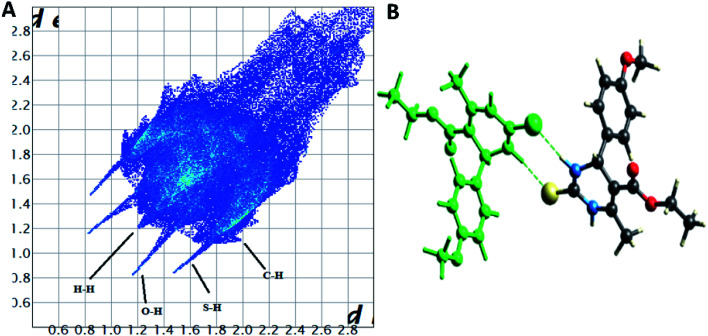
(A) Two-dimensional fingerprint plot for compound 1.3, (B) non-covalent interactions forming R^2^_2_ (8) ring motif.

**Fig. 8:**
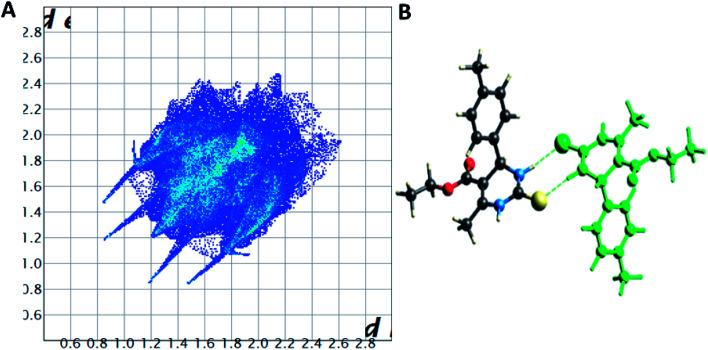
(A) Two-dimensional fingerprint plot for compound 1.4, (B) non-covalent interactions forming R^2^_2_ (8) ring motif.

**Fig. 9:**
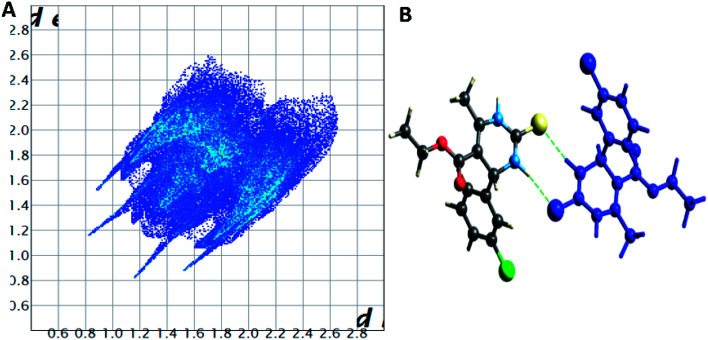
(A) Two-dimensional fingerprint plot for compound 1.5, (B) non-covalent interactions forming R^2^_2_ (8) ring motif.

The Hirshfeld surface analysis explores the intermolecular contacts and their role in crystal packing^31,32^ The Hirshfeld surface mapped on d_norm_ of compounds 1.3, 1.4, and 1.5 are displayed in Fig. S1(A), S2(A), and S3(A) (ESI†), where the red region indicates the weak interaction contacts with a shorter distance. In contrast, white and blue regions indicate weak interaction contacts with a distance longer and equal to the van der Waals radii, respectively (Fig. S1(A), S2(A), and S3(A)) (ESI†). The red color on the *d*_norm_ surface represents more dominant non-covalent C–H⋯O interactions in the crystal structure. The 2D fingerprint plot demonstrates the different combinations of de and di across the molecule's surface. In addition, it also represents the summary of weak intermolecular interactions in crystal structure and provides information about the percentage contribution of 3D Hirshfeld surface.^33^ The 2D fingerprint plots of compounds 1.3, 1.4, and 1.5 are shown in Fig. 7A, 8A and 9A, and percentage contributions of N⋯H, O⋯H, H⋯H, C⋯C, and C⋯H interactions are represented in a bar graph (Fig. S4) (ESI†). The Hirshfeld weak interactions calculation is in Table 3.

**Table 3:** Non-covalent interactions (Å) for 1.3, 1.4 & 1.5 in Hirshfeld energy calculations

Donor–H⋯acceptor	D–H, Å	H⋯A, Å	D⋯A, Å	D–H⋯A, ^o^	Symmetry operation
**Compound** **1.3**					
N3–H3⋯O0aa	1.205	1.981	2.983	171.78	*x*, *y*, *z*
N4–H4⋯S1	1.009	2.319	3.316	169.20	−*x* + 1/2, −y + 1/2, −*z*
C3–H3a⋯C2	1.083	2.725	3.581	142.65	−*x* + 1/2, *y* + 1/2, −*z* + 1/2

**Compound** **1.4**					
N2–H2⋯O0aa	1.009	2.061	3.058	169.06	*x*, *y*, *z*
N3–H3⋯S1	1.009	2.334	3.322	166.24	−*x*, −*y*, −*z*
C9–H9b⋯S1	1.083	2.993	4.046	164.18	−*x*, −*y*, −*z*

**Compound** **1.5**					
C1–H1⋯O0aa	1.083	2.604	3.634	158.51	−*x*, −*y*, −*z*
N3–H3⋯O0aa	0.504	2.981	4.189	168.82	*x*, *y*, *z*
C9–H9c⋯C13	1.083	2.728	3.754	158.08	*x*, *y*, *z*
C9–H9c⋯C14	1.083	2.750	3.797	162.65	*x*, *y*, *z*
N2–H2⋯S1	0.504	2.408	3.401	167.85	−*x*, −*y*, −*z*
C8–H8b⋯C10	1.083	2.676	3.672	152.66	−*x*, −*y*, −*z*

Spoke-like pattern in the fingerprint plots of 1.3, 1.4 and 1.5 represents C–H⋯O interactions in crystal lattice in region of di + de = 2.00–3.0 Å, with H⋯O/O⋯H contributions of 12.4%, 6.9% and 7.7%, respectively (Fig. 7A, 8A and 9A). The second spoke-like pattern in the fingerprint plots of 1.3, 1.4 and 1.5 represents C–H⋯S interactions in crystal lattice in region of di + de = 2.30–3.4 Å with H⋯S/S⋯H contributions of 15.1%, 15.5% and 16.7%, respectively (Fig. 7A, 8A and 9A). The C–H⋯π interactions in compounds 1.3, 1.4 and 1.5 can be seen as a pair of unique blue-colored wings in the region of di + de = 3.2–3.6 Å with H⋯C/C⋯H contribution of 15.2%, 14.2% and 12.9%, respectively (Fig. 7A, 8A and 9A). The C–H⋯N pair of contacts is also reflected as two characteristic wings occupied in di + de = 3.2–3.5 Å in compounds 1.3, 1.4, and 1.5. The yellowish-red bin is absent on the fingerprint plots in compounds 1.3 and 1.4, which means the absence of weak π⋯π stacking in crystal packing (Fig. 7A and 8A. However, there is a light yellowish-red bin (with C⋯C contact of contribution is 3.9%) present on the fingerprint plot indicates the existence of weak π⋯π stacking interactions between the phenyl rings in compound 1.5 (Fig. 9A). The Hirshfeld weak interactions calculation found a similar weak non-covalent intermolecular interactions pattern in crystal packing. C–H⋯π interactions, C–H⋯N, and C–H⋯O interactions, *etc.*, of compound 1.3, 1.4 and 1.5 in crystal packing structure are in Fig. 7B, 8B, 9B) and Table 3. In all three crystal structure's, 1.3, 1.4, and 1.5, extensive hydrogen-bonding network of calculated interactions, terminal carbonyl oxygen, thio-pyrimidine nitrogen, and thio-pyrimidine sulfur are involved in weak interaction and forming eight membered R^2^_2_ (8) (Fig. 7B, 8B and 9B) rings.

**Fig. 10:**
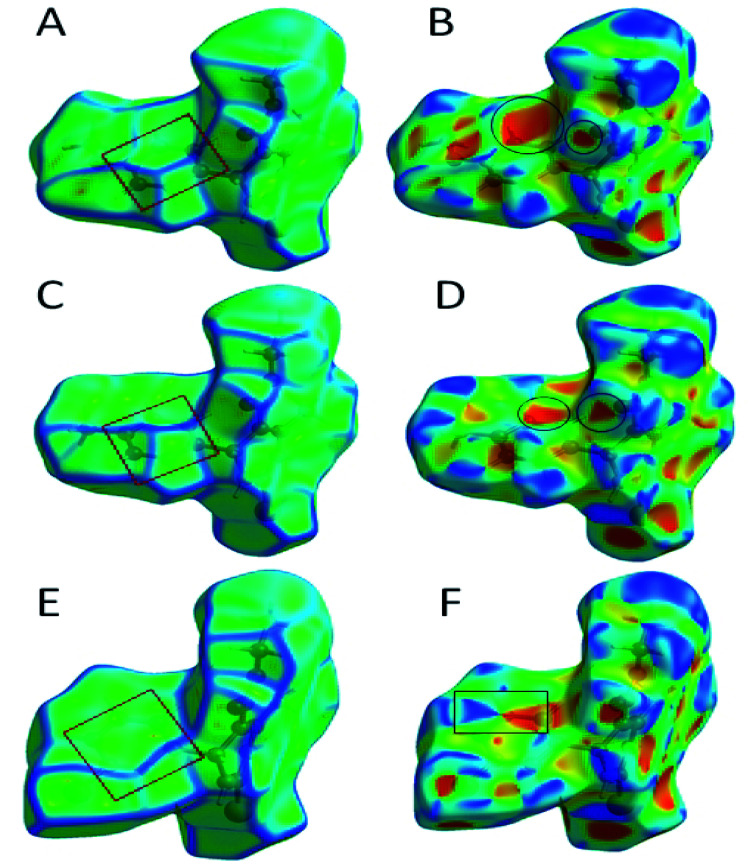
(A) Curvedness plot of compound 1.3, (B) shape index plot of compound 1.3, (C) curvedness plot of compound 1.4, (D) shape index plot of compound 1.4, (E) curvedness plot of compound 1.5, (F) shape index plot of compound 1.5.

The role of π⋯π stacking is central importance in chemistry as well as bio medicine field. They are key interactions influencing the tertiary structure of proteins, the vertical base stacking in DNA, and the intercalation of different drugs into DNA, which helps in drug design. The role of π⋯π stacking also become prominent in drug–receptor interactions as most of the drugs are aromatic, and about 20% of amino acids are aromatic. The curvedness plots and shape index plots of 3D Hirshfeld is a very promising tool to visualize π⋯π stacking interactions in the crystals.^34^ In curvedness plots, the yellow spots represent very weak intermolecular interactions, and red-yellow colored spots represent strong hydrogen-bonding interactions in the crystal structures of 1.3, 1.4, and 1.5 (Fig. 10). In curvedness plots of compounds 1.3 and 1.4, the absence of green-colored flat regions bounded by red-colored rectangles on the phenyl rings further confirms the non-existence of π⋯π stacking effect in crystal packing (Fig. 10A & C). However, in the curvedness plots of compound 1.5, flat green colored regions bounded by red-colored rectangles on phenyl rings, once again confirm the presence of π⋯π stacking effect in crystal packing of 1.5 (Fig. 10E). Red and blue regions represent the acceptor and the donor property, respectively, in the shape index of compound 1.3, 1.4, and 1.5 (Fig. 10B, D & F). Yellowish-red colored concave regions inside the counters indicate the presence of weak intermolecular interactions in the Shape index plots of compounds 1.3, 1.4, and 1.5 (Fig. 10B and D).The absence of adjacent red and blue triangles in the shape-index map of 1.3 and 1.4 also proved the lack of π⋯π stacking effect in the crystal packing (Fig. 10B and D). There are adjacent red and blue triangles bounded by the black colored rectangles in the shape-index map of 1.5, once again confirms the π⋯π stacking effect in the crystal packing of 1.5 (Fig. 10F).

### 3.3. Density functional theory (DFT) calculations for 1.3, 1.4 and 1.5

**Fig. 11:**
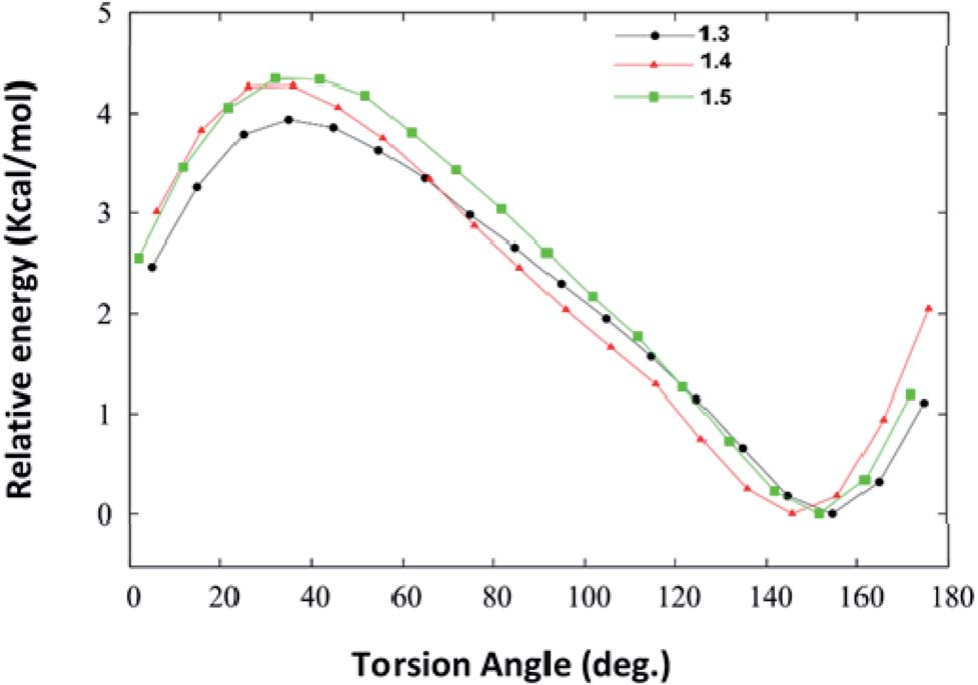
Potential energy surfaces for twisting the phenyl rings in the synthesized DHPM derivatives (1.3, 1.4, 1.5) as obtained at the M06-2X/6-31+G(d,p) level of theory.

We have also carried out the DFT calculations to know how the substituents impact the geometry of different synthesized DHPM derivatives and calculated the interaction energies of DHPM derivatives (1.3, 1.4 and 1.5) with their neighbouring molecules in the crystal structure at the M06-2X/6-31+G(d,p) level of theory. Torsional PES (potential energy surfaces) for twisting the phenyl rings in synthesized DHPM derivatives (1.3, 1.4 and 1.5) are shown in Fig. 11.

We note from Fig. 11 that each PES is characterized by only one minimum and the fully planar configurations (0° and 180°) are unstable. The DHPM derivatives with torsion angle (∼40°) are found to be energetically most unstable. The global minimum for 1.3, 1.4 and 1.5 molecule are found to be at torsion angle of 155.5°, 146.8° and 152.3°, respectively (Fig. 11), which is in agreement with their crystal structure. The optimized geometries of 1.3, 1.4 and 1.5 are shown in Fig. S5 (ESI†). Thus, our DFT calculations show that substituents do not impact the geometry of synthesized DHPM derivatives appreciably.

We also calculated the interaction energies of DHPM derivatives (1.3, 1.4 and 1.5) with their neighbouring molecules in the crystal structure at the M06-2X/6-31+G(d,p) level of theory. We considered neighbouring molecules which are in direct contact with a central molecule for complex formation and thus complexes formed by 8, 5 and 7 molecules for 1.3, 1.4 and 1.5, respectively, were taken for calculation of interaction energy. These complexes are shown in Fig. S7 and S8 (ESI†). The interaction energy of a complex was calculated as follows:Interaction energy = total energy of the complex − *n* × total energy of one moleculewhere *n* is the number of molecules in the complex.

The interaction energies of the complexes of 1.3, 1.4 and 1.5 are found to be −103.20, −24.61, −90.81 kcal mol^−1^, respectively. The interaction energy per molecule for 1.3, 1.4 and 1.5 are −12.90, −6.15 and −12.97 kcal mol^−1^, respectively. This indicates that 1.4 has very less tendency to associate with other molecule as compared to 1.3 and 1.5 molecule.

### 3.4. Molecular docking analysis of 1.1, 1.2, 1.3, 1.4, and 1.5

*In silico* analysis was carried out for compounds 1.1, 1.2, 1.3, 1.4, and 1.5 to study the interactions and binding energies of these compounds with kinesin Eg5 protein (PDB id: 3UIH). Pymol and Discovery studio visualizer was for the analysis of docking results.

**Fig. 12:**
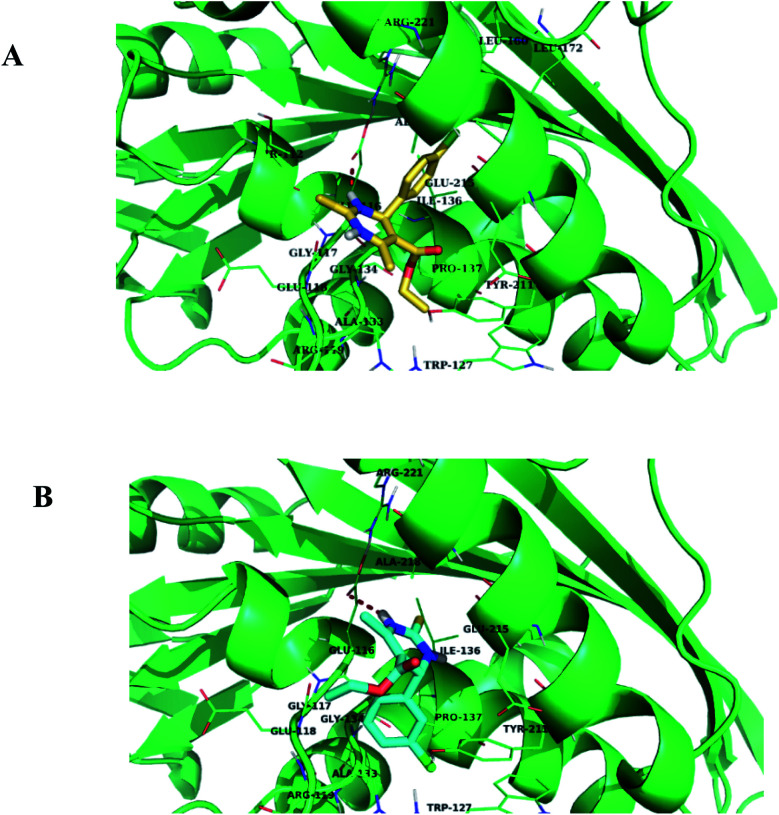
(A) and (B) Binding mode of compounds 1.2 and 1.5 in the active site cavity of Eg5 protein, respectively.

The common trend in the binding interactions of compounds 1.2, 1.5, and 1 (monastrol) in the cavity of Eg5 is observed. Ester group protrudes outside the cavity of Eg5. *In silico* analysis revealed that compounds 1.2 and 1.5 showed better binding energy (−7.9 kcal mol^−1^) than the standard drug monastrol (−7.8 kcal mol^−1^) (Table 4). Chlorobenzene ring of compounds 1.2 and 1.5 is directed towards the hydrophobic region of the active site of Eg5 (Fig. 12A and B). The chlorobenzene ring of 1.2 and 1.5 are orientated towards the hydrophobic region, so π-anion interactions with the negatively charged residue Glu116 will be favorable. Compound 1.5 also showed one halogen bond with the residue Trp127, which indicated that compound 1.5 fits nicely into the cavity of Eg5. Further, the residues Ala 133, Pro137, and Tyr211, Leu214, and Arg221 facilitate the hydrophobic π-alkyl and alkyl interactions with compound 1.5 (Fig. 12B) and (Table 4).

**Table 4:** Binding energy and the residues involved in the interaction of 1.1, 1.2, 1.3, 1.4, and 1.5 with kinesin Eg5 protein

Compounds	Docking score (kcal mol^−1^)	Residues involved in H-bond	Residues involved in other interactions (π-anion, π–σ, π–π, π-alkyl, and alkyl)
1.1	−7.8	Glu116	Ala 133, Pro 137
1.2	**−7.9**	Glu116	Leu160, Ile136, Leu214, Arg221, Phe239
1.3	−7.1	Glu116	Glu116, Arg119, Leu214, Ala218
1.4	−7.7	Glu116	Glu116, Ile136, Leu214, Phe239
1.5	**−7.9**	Glu116, Trp127 (halogen bond)	Ala 133, Pro137, Tyr211, Leu214, Arg221
1 (monastrol)	−7.8	Glu116, Glu118	Arg119, Ala133, Pro137, Leu214, Ala218

### 3.5. Cytotoxicity and anti-cancer activity of 1.1–1.5

#### 3.5.1. Compounds 1.1, 1.2, 1.3, 1.4, and 1.5 variants decreased lung adenocarcinoma A549 cells viability

**Fig. 13:**
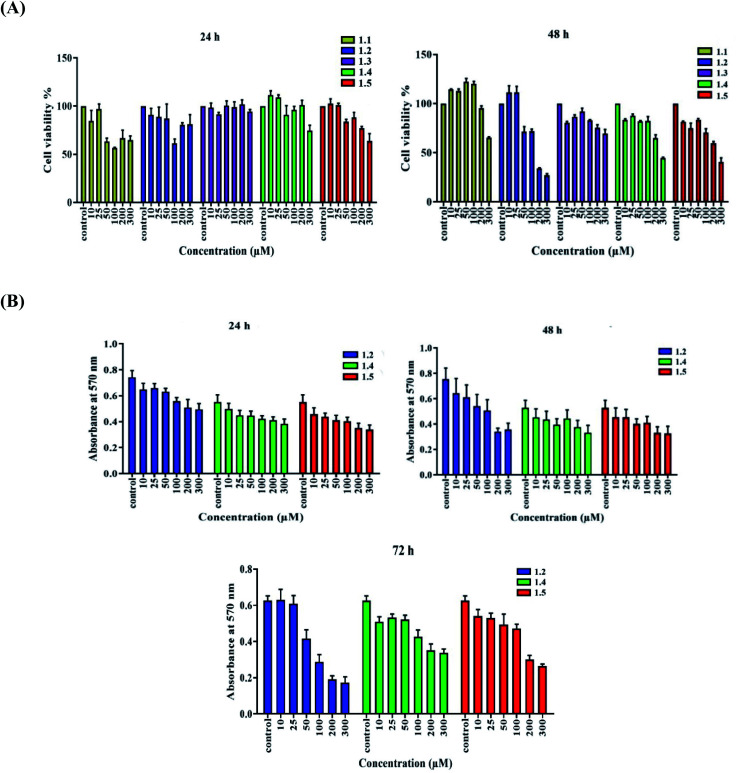
A549 lung adenocarcinoma cells were treated with (A) diverse concentrations (10, 25, 50, 100, 200, and 300 μM) of compounds (1.1, 1.2, 1.3, 1.4, and 1.5), and an MTT assay was done to test cell viability. (B) Varied concentrations of selected compounds 1.2, 1.4, and 1.5 were added for 24, 48, and 72 h, and an MTT assay was done.

A549 adenocarcinoma cells were treated at different concentrations (10–300 μM) of compound variants (1.1, 1.2, 1.3, 1.4 and 1.5) to screen the highly effective variant. 1.1 treatment (10–300 μM) decreased cell viability to 3–43.47% at 24 h and 4.6–34.8% at 48 h (Fig. 13A). 1.3 treatment (10–300 μM) decreased cell viability to 0–8.34% at 24 h and 8–30.7% at 48 h (Fig. 13A). 1.2, 1.4, and 1.5 (10–300 μM) treatments decreased cell viability to 13–33.3%, 10–30.7% and 17–38.7%, respectively after 24 h. After 48 h treatment (10–300 μM), 1.2, 1.4, and 1.5 decreased cell viability to 14–55%, 17–38.7% and 14–38.3%, respectively. After 72 h time point, 1.2, 1.4, and 1.5 decreased cell viability to 3–72.5%, 15.1–46.2% and 14–58%, respectively (Fig. 13B). Among this series of variant 1.2, 1.4, and 1.5 decreases the cell viability significantly.

#### 3.5.2. Compounds 1.1, 1.2, 1.3, 1.4, and 1.5 increased cell death in A549 cells

**Fig. 14:**
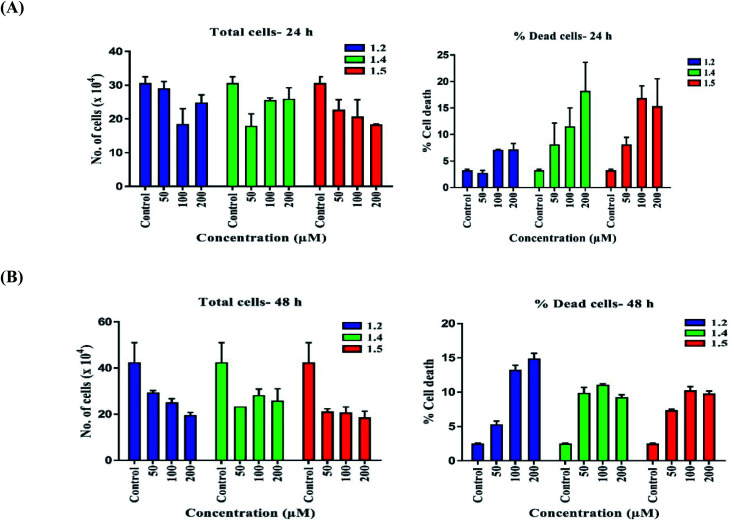
Trypan blue assay was performed to check the effect of 1.2, 1.4, and 1.5 at 50, 100, and 200 μM doses. Graphs represent percent cell death and the total number of cells for (A) 24 h and (B) 48 h.

Trypan blue assay was performed to check cell growth inhibition and cell death. Lung adenocarcinoma cells were treated with 1.2, 1.4, and 1.5 at defined doses (50, 100, and 200 μM) for 24 and 48 h. In 1.2 treated groups, 7.18%, in 1.4 treated groups 18.22%, and in 1.5 treated groups, 16.88% cell death was observed at 24 h (Fig. 14A) in the 200 μM treatment group. At 48 h time point, 14.92%, 11.07%, and 10.27% cell death was observed in 1.2, 1.4, and 1.5 treated groups, respectively (Fig. 14B). The number of total cells decreased as the concentration of variants was increased compared to control groups at 24 h (Fig. 14A). At 200 μM concentration, 1.2 treatment caused the highest cell death at 48 h, and 1.2 and 1.5 treatments decreased total cell number to less than 50% at 48 h (Fig. 14B). These results suggest that treatment with 1.2, 1.4, and 1.5 compounds induced significant cell death in A549 cells. We chose 1.2 and 1.5 compounds for further study as the percentage of cell death was higher in these groups after treatment.

#### 3.5.3. Compounds 1.2 and 1.5 induced apoptosis in A549 cells

**Fig. 15:**
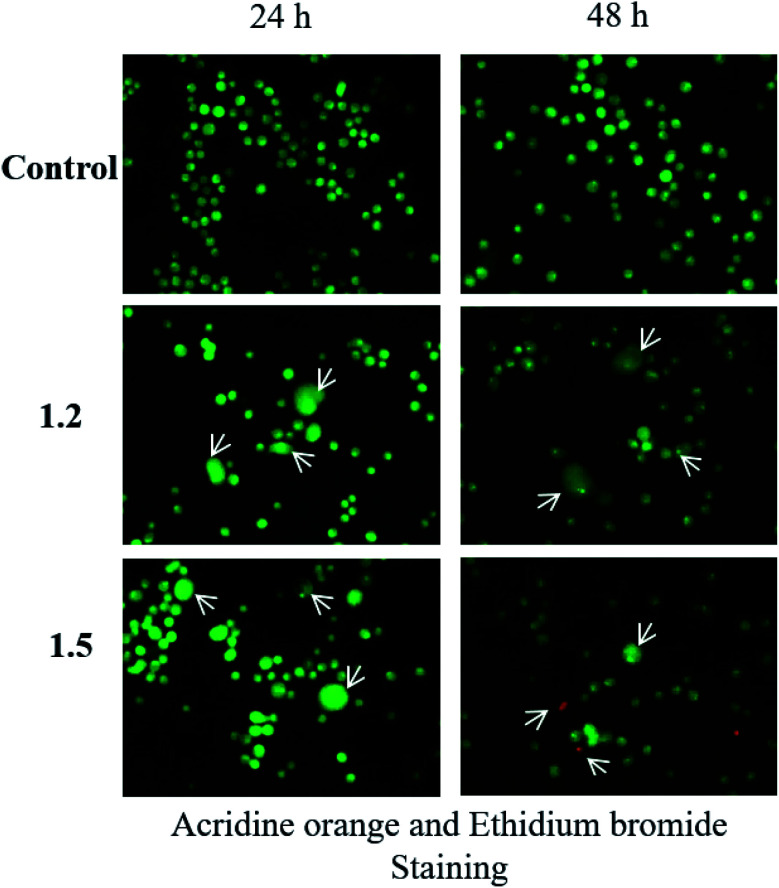
The lung adenocarcinoma cells were treated with 200 μM 1.2 and 1.5 compounds for 24 and 48 h and stained as described in methods (*n* = 3).

The compounds 1.2 and 1.5 were selected for studying apoptosis induction in lung adenocarcinoma cells because the percentage of cell death was higher with these two analogues. Acridine orange-ethidium bromide staining distinguishes live, apoptotic, and necrotic cells. Live cells appear green, and dead cells appear red. The control cells did not show any characteristics of cell death at any time point. Treatment with 1.2 and 1.5 compounds at 200 μM for 24 and 48 h showed typical apoptotic characteristics. Membrane blebbing, apoptotic body formation and nuclear fragmentation were observed at 24 and 48 h post-treatment. Treatment with 1.5 for 48 h caused necrosis in cells that appeared red (Fig. 15). These results suggest that 1.2 and 1.5 compound treatments induced cell death in lung adenocarcinoma cells.

#### 3.5.4. Compounds 1.2 and 1.5 induced nuclear fragmentation A549 adenocarcinoma cells

**Fig. 16:**
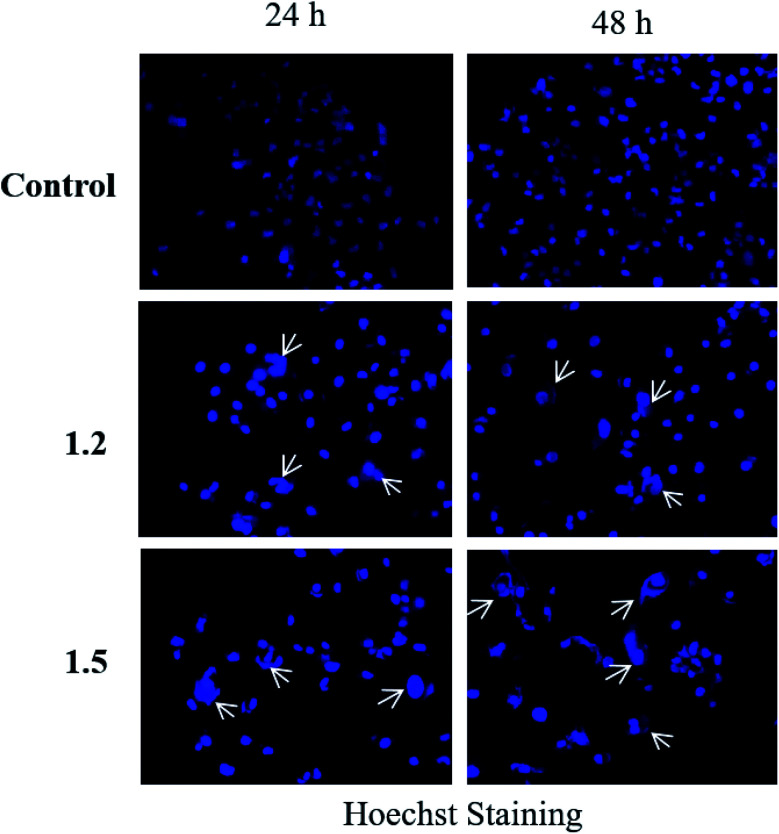
A549 adenocarcinoma cells were treated with 200 μM 1.2 and 1.5 compounds for 24 and 48 h and stained with Hoechst stain. Representative images are shown (*n* = 3) at 200× magnification.

Hoechst assay was performed to analyze the effect of 1.2 and 1.5 compounds on apoptosis of A549 cells. Control cells did not show any characteristics associated with cell death. In contrast, in 1.2 and 1.5 (200 μM) treated groups, DNA fragmentation, nuclear condensation, and apoptotic body formation were observed by bright blue fluorescence after 24 and 48 h of treatment (Fig. 16). These results suggest that 1.2 and 1.5 treatments could induce apoptosis in A549 cells.

It has been observed that DHPM exists as a dimer as found in all 1.3, 1.4, and 1.5 crystal structures. Our results through the single crystal and Hirshfeld analysis found the existence of the dimeric structure of DHPM. The two such monomers lead to the dimer by forming an additional hydrogen bond yielding the bifurcated hydrogen bonds and N–H–S bridges. The presence of the thioamide group in the series exists in monomer–dimeric equilibrium. The two monomers lead to the dimer by forming by N–H–S hydrogen bond.

In addition to the N–H–S hydrogen bond (Figs. 4, 5, and 6), two more intermolecular hydrogen bonding interactions C–H⋯O, N–H⋯O are observed between the oxygen atom of the ester group and hydrogen atom of the thioamide group. These additional H-bonds form tetramer of DHPM. The overall structure of the tetramer is close to planar. Hydrogen bonding represents one of the most versatile interactions that could be used for molecular recognition. It might be possible to interfere with the strength of the hydrogen bond effectively by linking the hydrogen-bonding site to a binding site of the molecule with the receptor. The presence of π⋯π stacking in compound 1.5 will also help to study the drug receptor interactions of this compound.

A comparision of *in vitro* effects of synthesized DHPM derivatives (1.2 and 1.5) with potent anticancer agents such as monastrol, dimethylenastron, compounds 1, 2, and 3 (Fig. 2) from DHPM scaffold is shown in Table 5. Monastrol and dimethylenastron inhibit mitotic kinesin Eg5 protein, whereas compound 1 inhibit proliferation of prostate cancer cells PC3 (IC_50_ = 37 μM) as well as lung cancer cells NCI–H1299 (IC_50_ = 40 μM). Compound 2 inhibit proliferation of leukemia cell lines HL-60(TB) (IC_50_ = 0.05656 μM) as well as MOLT-4 (IC_50_ = 1.788 μM). Compound 3 also inhibit proliferation of breast cancer cell lines SK-BR-3 with GIC_50_ value of ±0.4 μM. Whereas, synthesized DHPM derivatives 1.2 and 1.5 significantly inhibit proliferation of lung adenocarcinoma cells A549 at 200 μM concentration.

**Table 5:** *In vitro* effects of synthesized DHPM derivatives (1.2 and 1.5) with related anticancer drugs

Anticancer drugs	Target with efficiency
Monastrol	Inhibit mitotic kinesin Eg5 protein (IC_50_ = 14 μM)
Dimethylenastron	Inhibit mitotic kinesin Eg5 protein(IC_50_ = 200 nM)
Compound 1	Inhibit proliferation of prostate cancer cells PC3 (IC_50_ = 37 μM), lung cancer cells NCI–H1299 (IC_50_ = 40 μM)
Compound 2	Inhibit proliferation of leukemia cell lines HL-60(TB) (IC_50_ = 0.05656 μM) and against MOLT-4 (IC50 = 1.788 μM)
Compound 3	Inhibit proliferation of breast cancer cell lines SK-BR-3 (GIC_50_ = ±0.4 μM)
1.2	Significantly inhibit proliferation of lung adenocarcinoma cells A549 at 200 μM
1.3	Significantly inhibit proliferation of lung adenocarcinoma cells A549 at 200 μM

Both *in silico* and *in vitro* results also found the same order of anti-cancer activity. An *in silico* study showed that order of binding affinity is 1.2–1.5 > 1.1 > 1.4 > 1.3, whereas *in vitro* study showed that variants 1.2, 1.4, and 1.5 decreased the cell viability significantly. Analogues 1.2, 1.4, and 1.5 also induced significant cell death in A549, but 1.2 and 1.5 were found significant in this group. Both 1.2 and 1.5 treatment caused apoptosis-induced cell death in A549 cells. Nuclear fragmentation in A549 adenocarcinoma cells by Hoechst staining showed that both 1.2 and 1.5 can induce apoptosis in cells.

As shown in tables 1.4, the values of inhibition zone for ligands are related to the nature of substituent as they increase according to the following order: *p*-Cl ∼ *m*-Cl > H > *p*-CH_3_ > *p*-OCH_3_. This can be attributed to the fact that the effective charge experienced by the central ring increased due to de-activating substituent (*p*-Cl ∼ *m*-Cl). At the same time, it decreased by activating group H > *p*-CH_3_ > *p*-OCH_3_. It is important to note that existence of a methyl and/or methoxy group enhances the electron density in the ring and simultaneously decreases inhibition zone values.

Electron withdrawing bridge would be expected to increase the acidity of proton donors and hence increase its binding ability as the electron-withdrawing character of a chloro group is relevant to the best analogue 1.2 and 1.5. In general, hydrogen bonds involving OH groups are proton donors, and their O atoms are proton acceptors. Both intra and intermolecular OH–N may form several structures in simultaneous equilibrium.

The DHPM series of compounds can induce intracellular oxidation resulting in cytosolic toxicity and genome toxicity in cancer cells. These events can further cause changes at the molecular level, ultimately resulting in an altered cell cycle leading to apoptosis. These events could also reduce migration ability and impede tumor growth resulting in reduced tumor size and migration of cancer cells. These findings suggest that 1.2 and 1.5 might be potential anticancer drug candidates. Thus, these analogues can be promising agents for treating various cancers, including lung cancer.

## 4. Conclusion

In summary, we elaborated green syntheses of dihydropyrimidinones using *Citrus macroptera* juice in a solvent-free reaction and deduced the primary SAR of anti-cancer. The advantages of this procedure include good yield, short reaction time, and eco-friendly procedure. The meaningful SARs can be drawn as follow: (i) deactivating group (Chlorine) at 3, 4 positions in the substituted ring of DHPM enhance activity; (ii) activating group in DHPM scaffold not suitable for active drug towards cancer cell. Moreover, we have studied the aromatic interactions using the single-crystal X-ray diffraction (SCXRD) method and the Hirshfeld surface analysis method. We found that C–H⋯π, C–H⋯O, C–H⋯N, C–H⋯C, lone pair⋯π, π⋯π interactions for synthesized compounds were beneficial for drug design, stabilization of DNA structure, protein folding, crystal engineering, material science, *etc.* Hirshfeld's surface analysis showed various intermolecular interactions in the crystal structures supporting the single-crystal X-ray diffraction (SCXRD) study. DFT calculations show that substituent does not impact the geometry of synthesized DHPM derivatives and conformational study of molecule reveals that it can change its conformation to get best interaction with active site. By these studies, exposure of different groups in analogues in the solid-state provides a clue for further study for biological activity concerning different receptors. As far as cytotoxicity and anticancer activity of these compounds are concerned, we found that they showed good anticancer activity. The anticancer activity was carried out in Human lung adenocarcinoma (A549) cells, where two derivative compounds (1.2 and 1.5) from Scheme 1 showed anticancer activity. The anticancer activity of these compounds (1.2 and 1.5) was well supported by *in silico* analysis, where they showed better binding energy than standard drug monastrol. Compound 1.4 showed poor anticancer activity compare to 1.2 and 1.5 molecules due to weak tendency of weak interactions as per finding in DFT calculation. Thus, anticancer activities suggest that our synthesized compounds could be potential candidates to develop anticancer drugs.

## Conflicts of interest

There are no conflicts to declare.

## Supplementary Material

RA-011-D1RA03969E-s001

RA-011-D1RA03969E-s002
