# Supplementary material for: Green synthesis, structural analysis and anticancer activity of dihydropyrimidinone derivatives
Source: RSC Adv. 2021 Nov 4;11(57):35737–53. doi: 10.1039/d1ra03969e (PMC9043124; doi:10.1039/d1ra03969e)
Supplement: RA-011-D1RA03969E-s001 [file RA-011-D1RA03969E-s001.pdf]

Electronic Supplementary Material (ESI) for RSC Advances.
This journal is © The Royal Society of Chemistry 2021

Supporting Information

for

Green Synthesis, Structural analysis and anticancer activity of dihydropyrimidinones derivatives

Hirshfeld surface analysis of compounds 1.3, 1.4 and 1.5. Density Functional Theory (DFT) Calculations of compounds 1.3, 1.4 and 1.5. NMR spectra of compounds 1.3, 1.4 and 1.5. CheckCIF of compounds 1.3, 1.4 and 1.5.

1.Hirshfeld Caculation and surface analysis of Compounds 1.3, 1.4 and 1.5

Table S1. Hirsfield Calculation for compound **1.3**

N	Symmetry operator	R	Electron Density	E_ele	E_pol	E_dis	E_rep	E_tot
1	-x, -y, -z	9.26	B3LYP/6-31G(d,p)	-17.5	-5.7	-17.0	22.8	-23.5
2	x, y, z	7.33	B3LYP/6-31G(d,p)	-35.0	-10.5	-22.1	38.2	-40.4
3	-x+1/2, y+1/2, -z+1/2	8.10	B3LYP/6-31G(d,p)	-14.2	-3.6	-39.8	24.5	-37.3
4	-x+1/2, -y+1/2, -z	8.37	B3LYP/6-31G(d,p)	-73.6	-15.5	-20.6	96.0	-47.9
5	x+1/2, y+1/2, z	9.83	B3LYP/6-31G(d,p)	-9.8	-2.4	-22.8	16.3	-21.9
6	-x, -y, -z	6.84	B3LYP/6-31G(d,p)	-13.2	-4.0	-43.7	19.4	-43.0
7	-x, y, -z+1/2	9.07	B3LYP/6-31G(d,p)	1.7	-0.5	-10.1	2.1	-6.1
8	x+1/2, y+1/2, z	9.83	B3LYP/6-31G(d,p)	-1.0	-0.5	-12.7	6.0	-8.8

Table S2. Hirsfield Calculation for compound **1.4**

N	Symmetry operator	R	Electron Density	E_ele	E_pol	E_dis	E_rep	E_tot
1	x, y, z	7.36	B3LYP/6-31G(d,p)	-32.0	-9.4	-22.0	33.4	-39.3
2	-x, -y, -z	9.08	B3LYP/6-31G(d,p)	-17.4	-5.4	-15.6	22.7	-21.9
3	-x, -y, -z	7.27	B3LYP/6-31G(d,p)	-4.3	-2.2	-30.8	15.0	-23.8
4	x, y, z	9.78	B3LYP/6-31G(d,p)	-0.1	-0.5	-12.8	6.0	-7.9
5	-x, -y, -z	8.37	B3LYP/6-31G(d,p)	-73.5	-15.5	-20.5	93.5	-49.3
6	-x, -y, -z	13.15	B3LYP/6-31G(d,p)	-0.4	-0.1	-2.4	0.0	-2.6
7	x, y, z	9.46	B3LYP/6-31G(d,p)	-10.8	-2.8	-25.7	17.6	-25.0
8	-x, -y, -z	6.11	B3LYP/6-31G(d,p)	-12.9	-4.7	-47.4	23.8	-43.7
9	-x, -y, -z	8.49	B3LYP/6-31G(d,p)	-4.1	-2.2	-39.8	17.7	-29.6
10	-x, -y, -z	9.10	B3LYP/6-31G(d,p)	0.4	-0.4	-12.9	5.3	-7.8

Table S3. Hirsfield Calculation for compound **1.5**

N	Symmetry operator	R	Electron Density	E_ele	E_pol	E_dis	E_rep	E_tot
1	-x, -y, -z	6.98	B3LYP/6-31G(d,p)	-21.8	-2.9	-53.0	42.5	-45.2
2	x, y, z	7.31	B3LYP/6-31G(d,p)	-33.6	-9.6	-22.0	34.7	-40.3
3	x, y, z	10.77	B3LYP/6-31G(d,p)	-3.5	-0.5	-9.9	9.2	-7.0
4	x, y, z	10.47	B3LYP/6-31G(d,p)	-8.2	-1.5	-17.4	14.2	-16.2
5	-x, -y, -z	6.11	B3LYP/6-31G(d,p)	-9.3	-4.1	-41.1	23.3	-34.3
6	-x, -y, -z	9.44	B3LYP/6-31G(d,p)	2.4	-0.8	-16.6	5.9	-8.9
7	-x, -y, -z	7.92	B3LYP/6-31G(d,p)	-70.5	-15.1	-21.7	80.3	-55.0
8	-x, -y, -z	6.86	B3LYP/6-31G(d,p)	-19.1	-4.8	-54.5	31.0	-52.1
9	-x, -y, -z	9.69	B3LYP/6-31G(d,p)	-7.0	-3.8	-13.1	6.1	-17.9

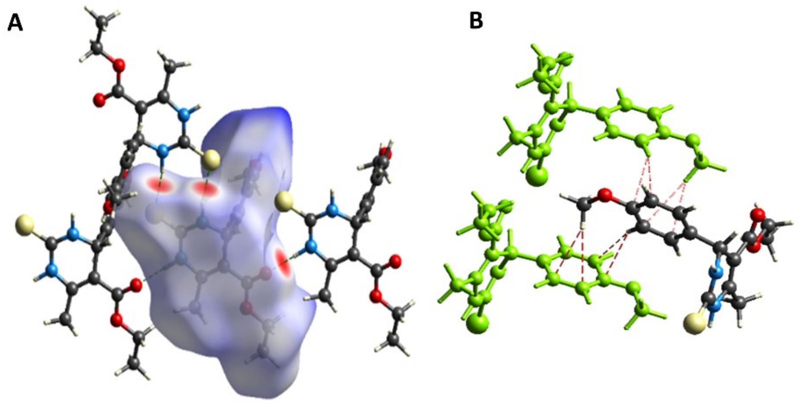

Figure S1: (A) Hirshfeld surface mapped on d_{norm} for compound **1.3**, (B) CH...π interactions in weak-interactions calculations of **1.3**

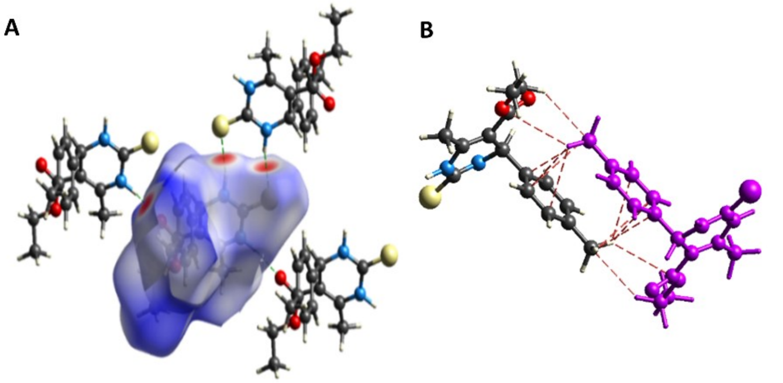

Figure S2: (A) Hirshfeld surface mapped on d_{norm} for compound **1.4**, (B) (D) C-H...π and C-H...O interactions in weak interactions calculations of **1.4**

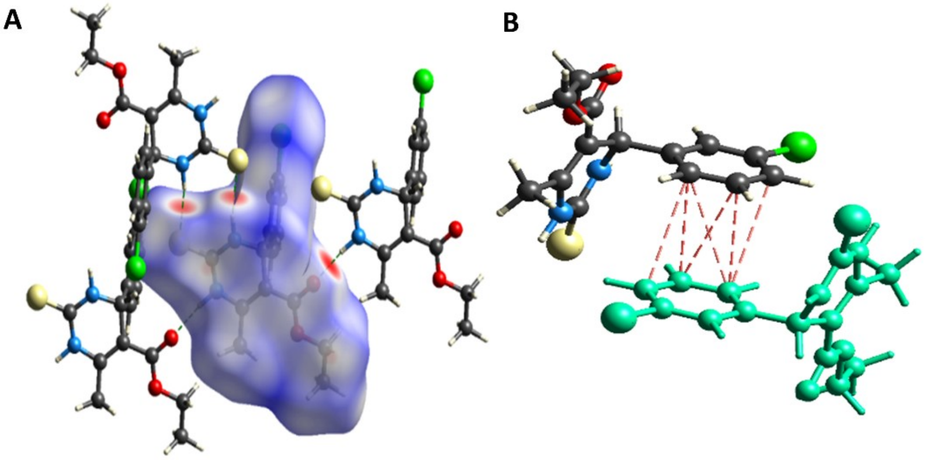

Figure S3: (A) Hirshfeld surface mapped on d_{norm} for compound **1.5**, (B) π...π interactions in weak interactions calculations of **1.5**

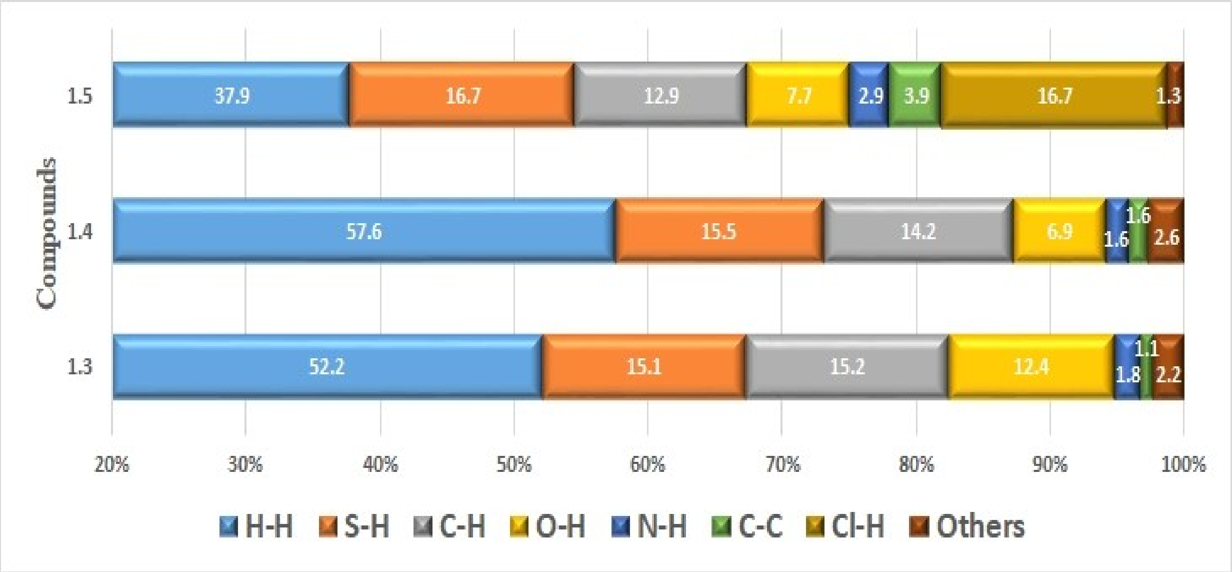

Figure S4: Percentage contributions in the fingerprint in compounds **1.3**, **1.4**, and **1.5** are shown in the bar graph.

2. Density Functional Theory (DFT) Calculations of compounds 1.3, 1.4 and 1.5

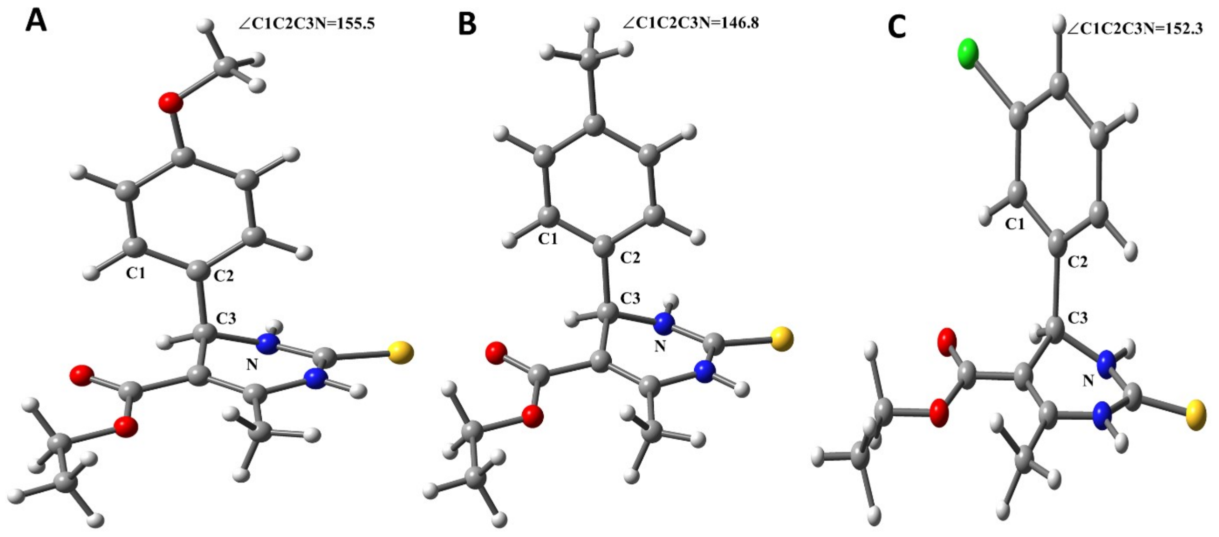

Figure S5: (A), (B) and (C) Optimized geometries of DHPM derivatives (**1.3, 1.4 and 1.5, respectively)** along with torsion angle, as obtained at the M06-2X/6-31+G(d,p) level of theory.

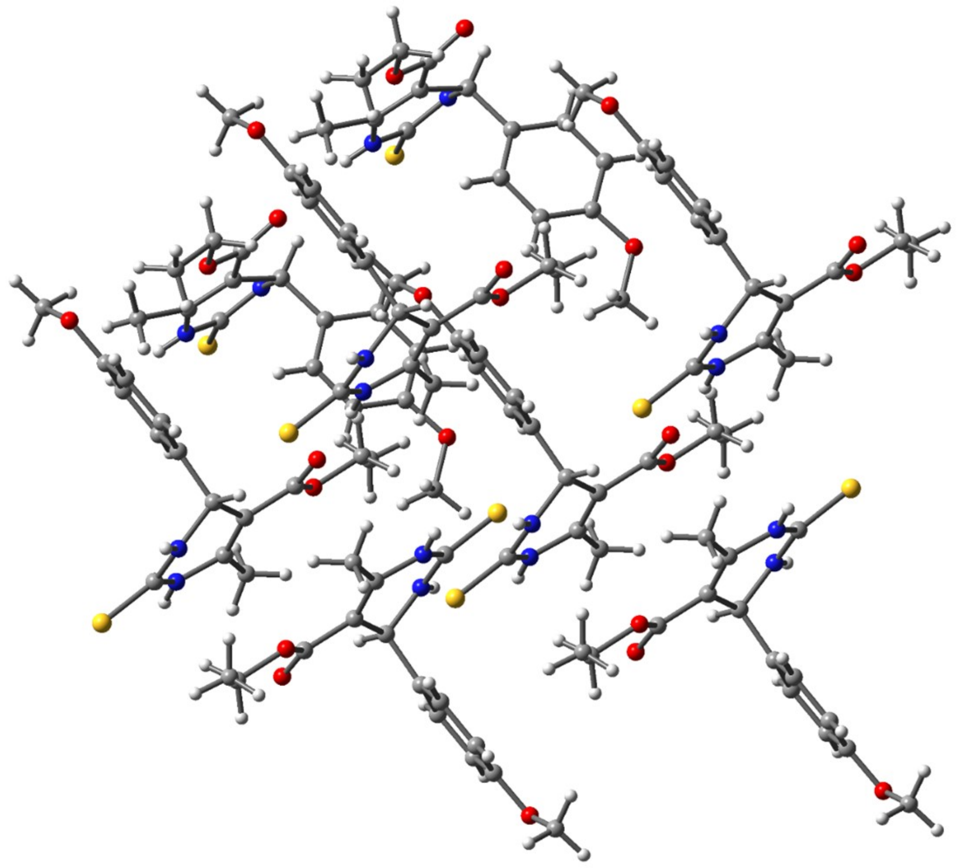

Figure S6: Structure of complex formed by 8 molecules of **1.3**.

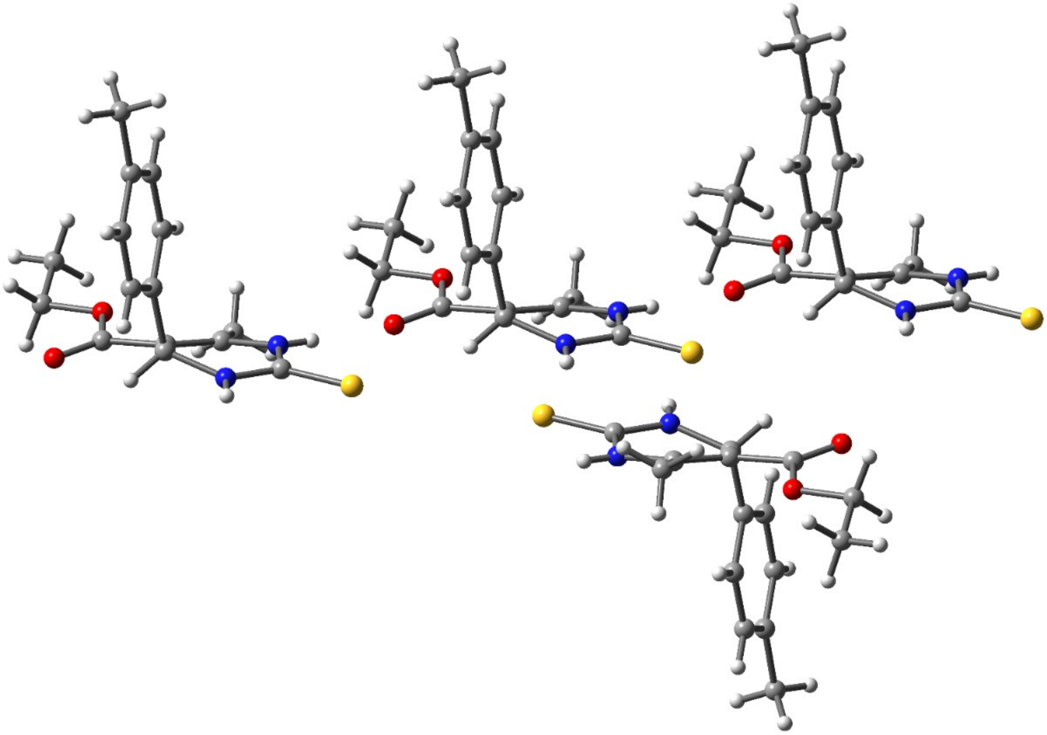

Figure S7: Structure of complex formed by 4 molecules of **1.4**.

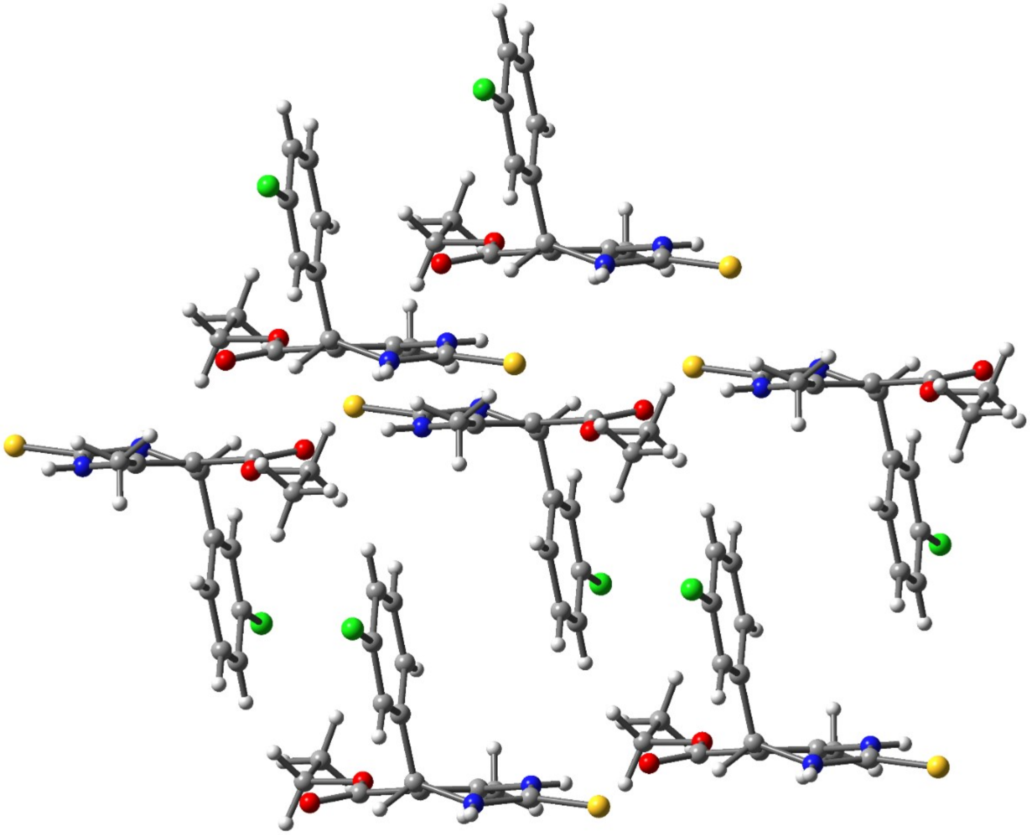

Figure S8: Structure of complex formed by 7 molecules of **1.5**.

3. NMR spectra of Compounds 1.1-1.5.

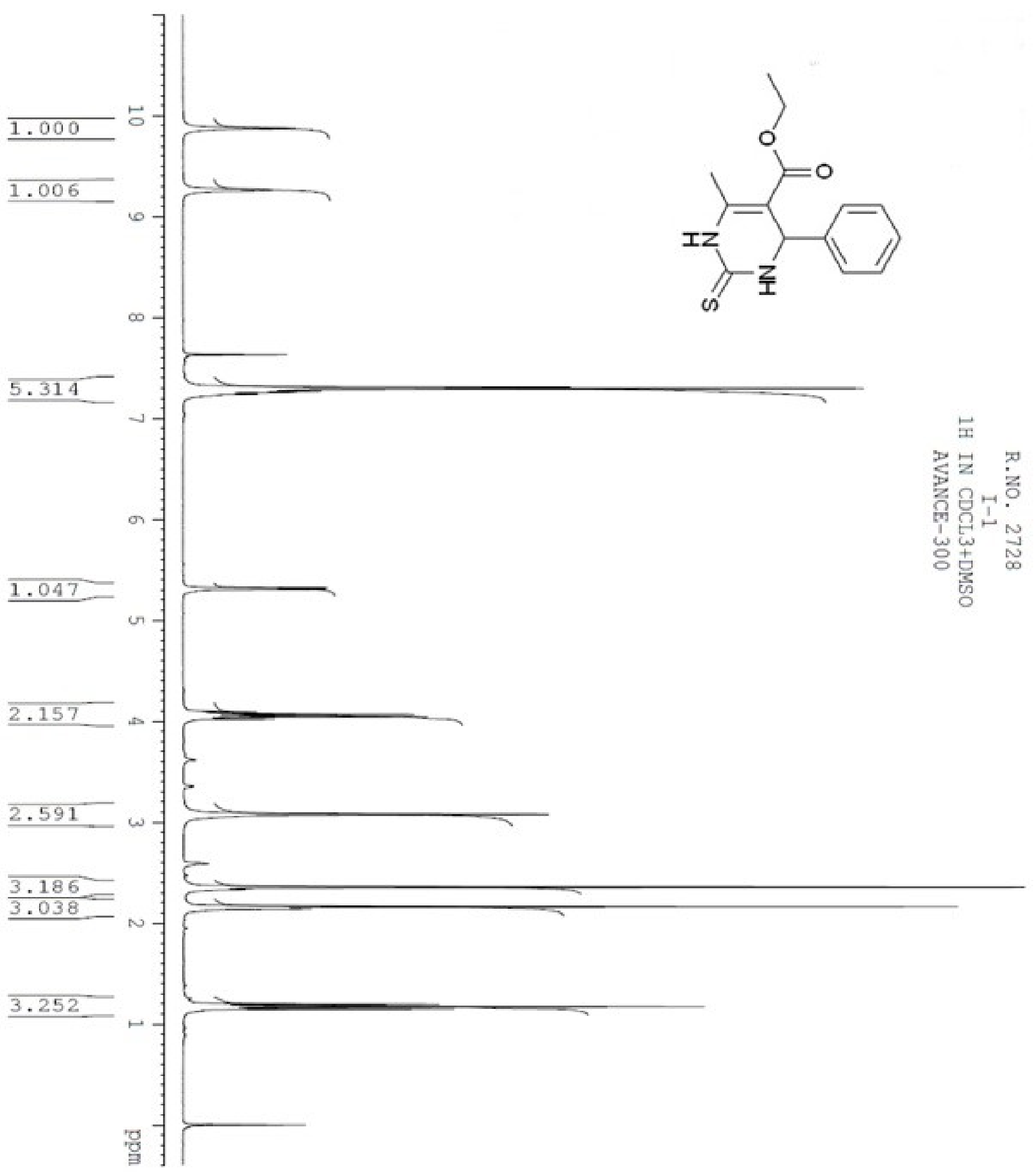

Figure S9: ^{1}H NMR of Compound **1.1**

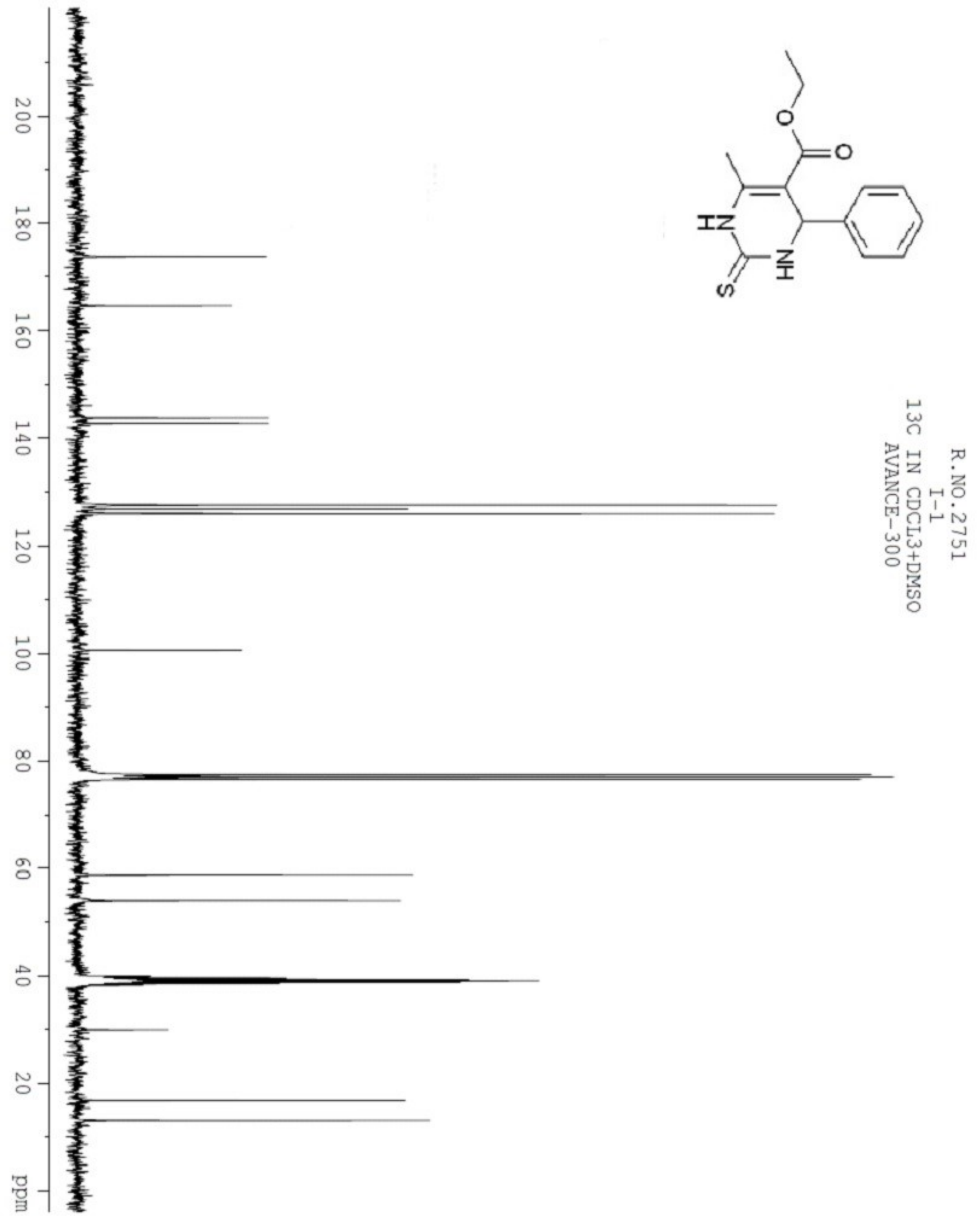

Figure S10: ^{13}C NMR of Compound **1.1**

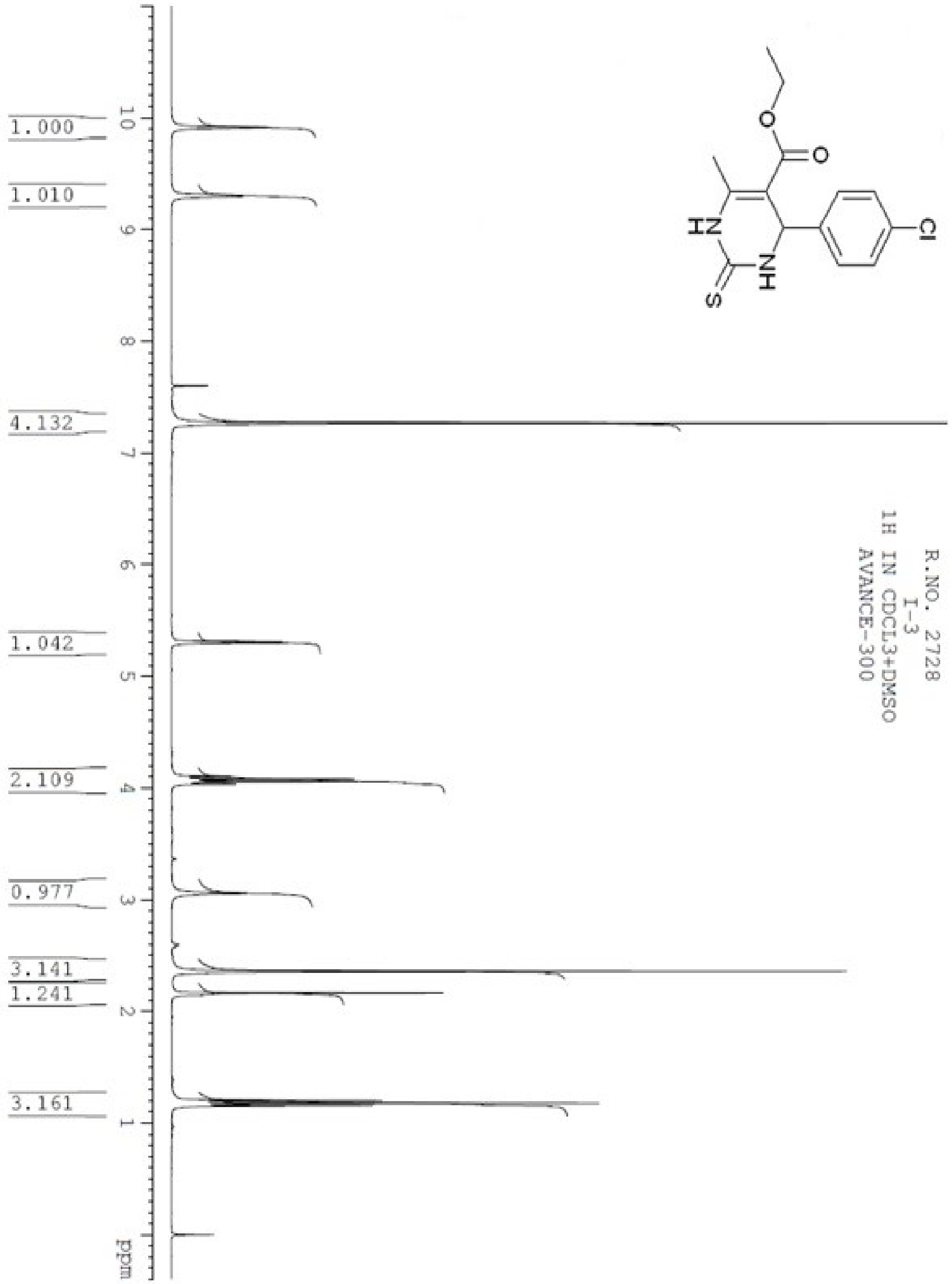

Figure S11 ^{1}H NMR of Compound **1.2**

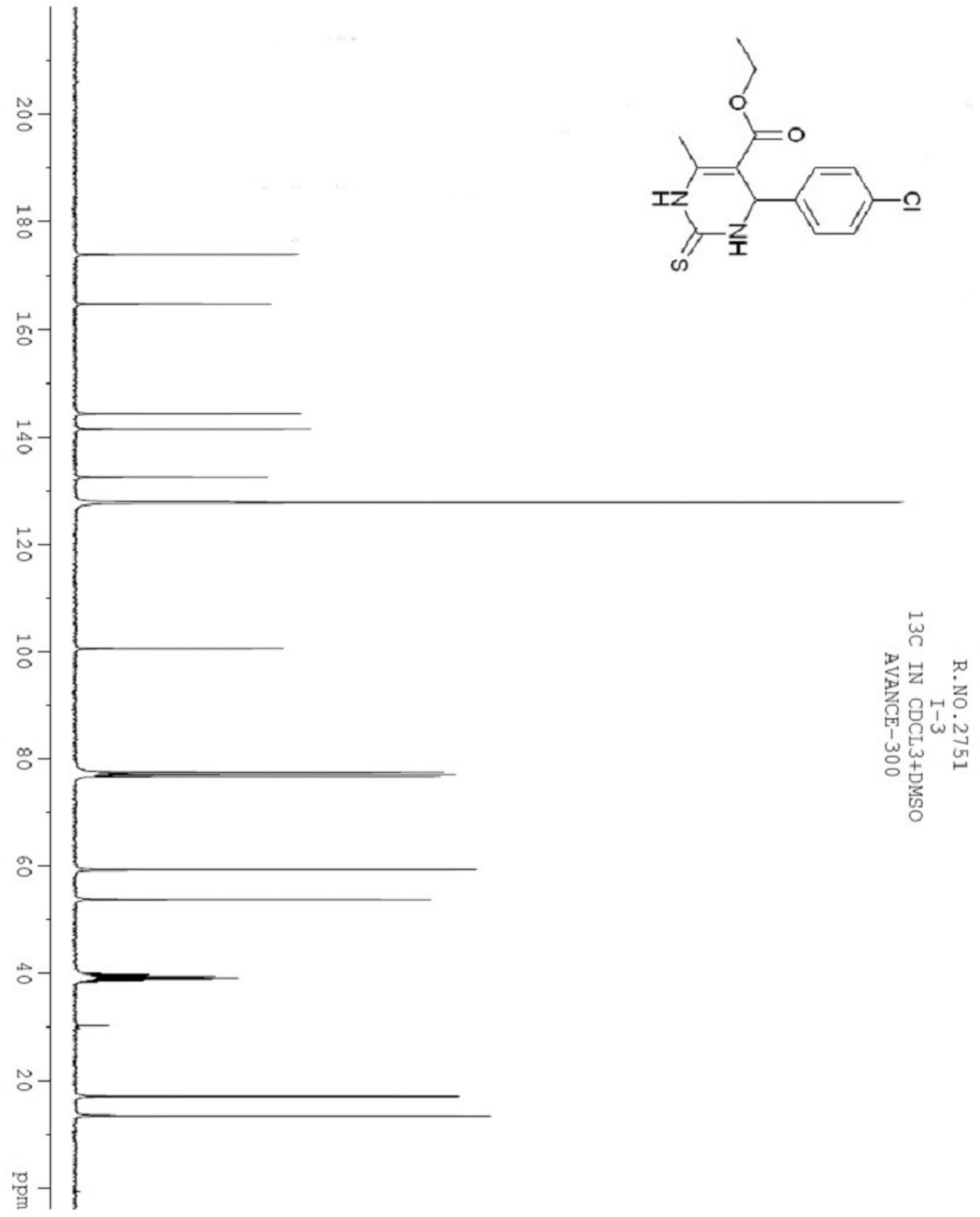

Figure S12: ^{13}C NMR of Compound **1.2**

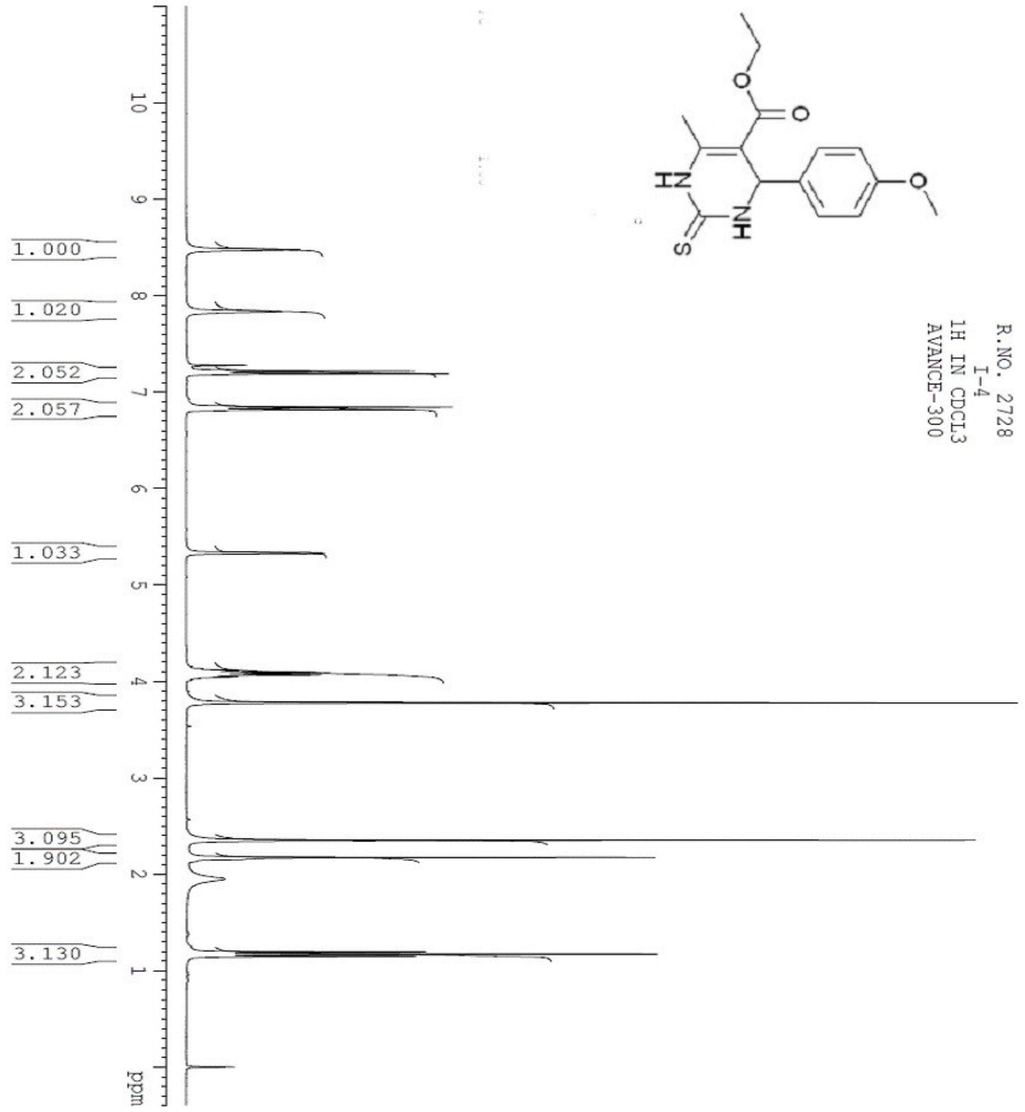

Figure S13: ^{1}H NMR of Compound **1.3**

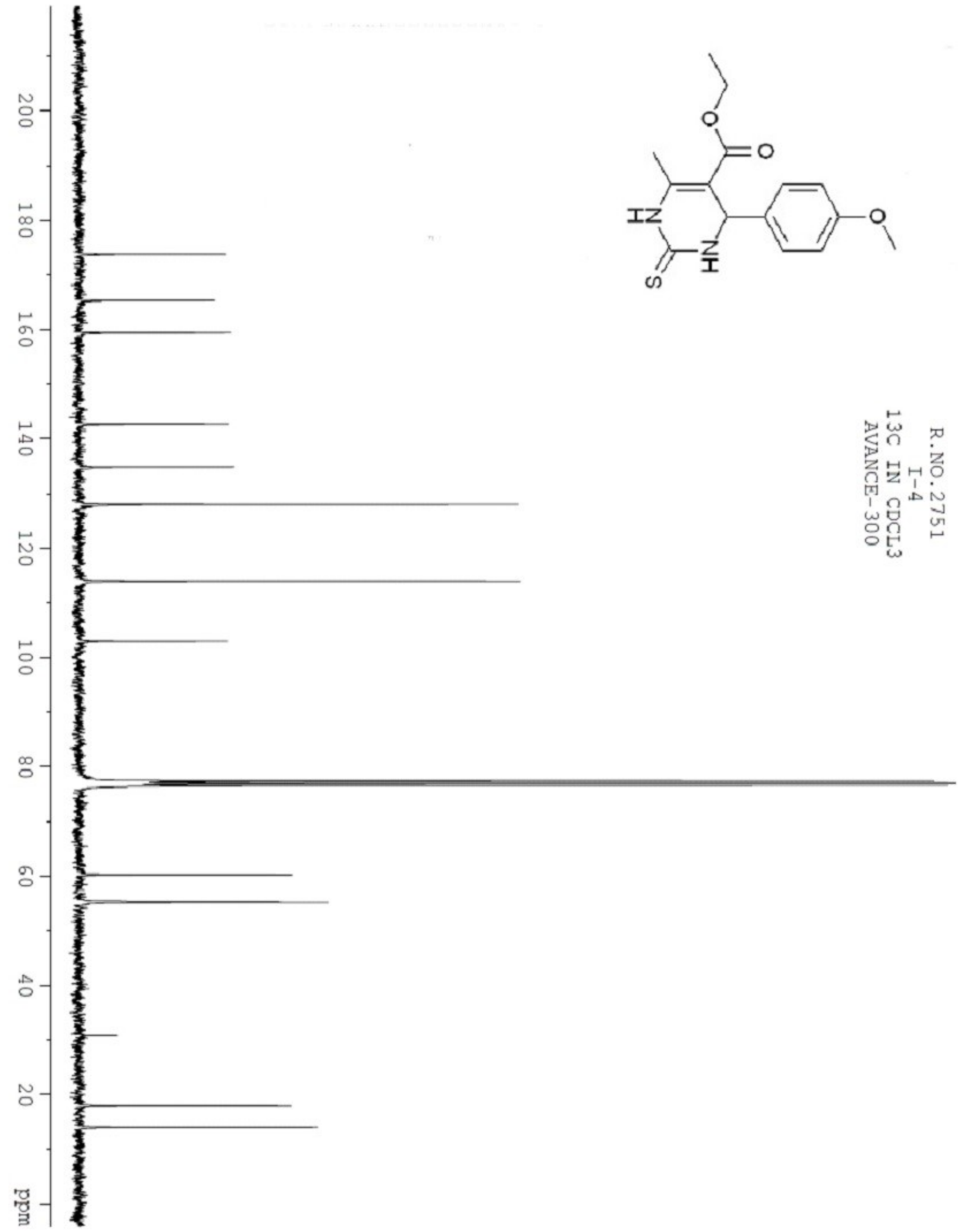

Figure S14: ^{13}C NMR of Compound **1.3**

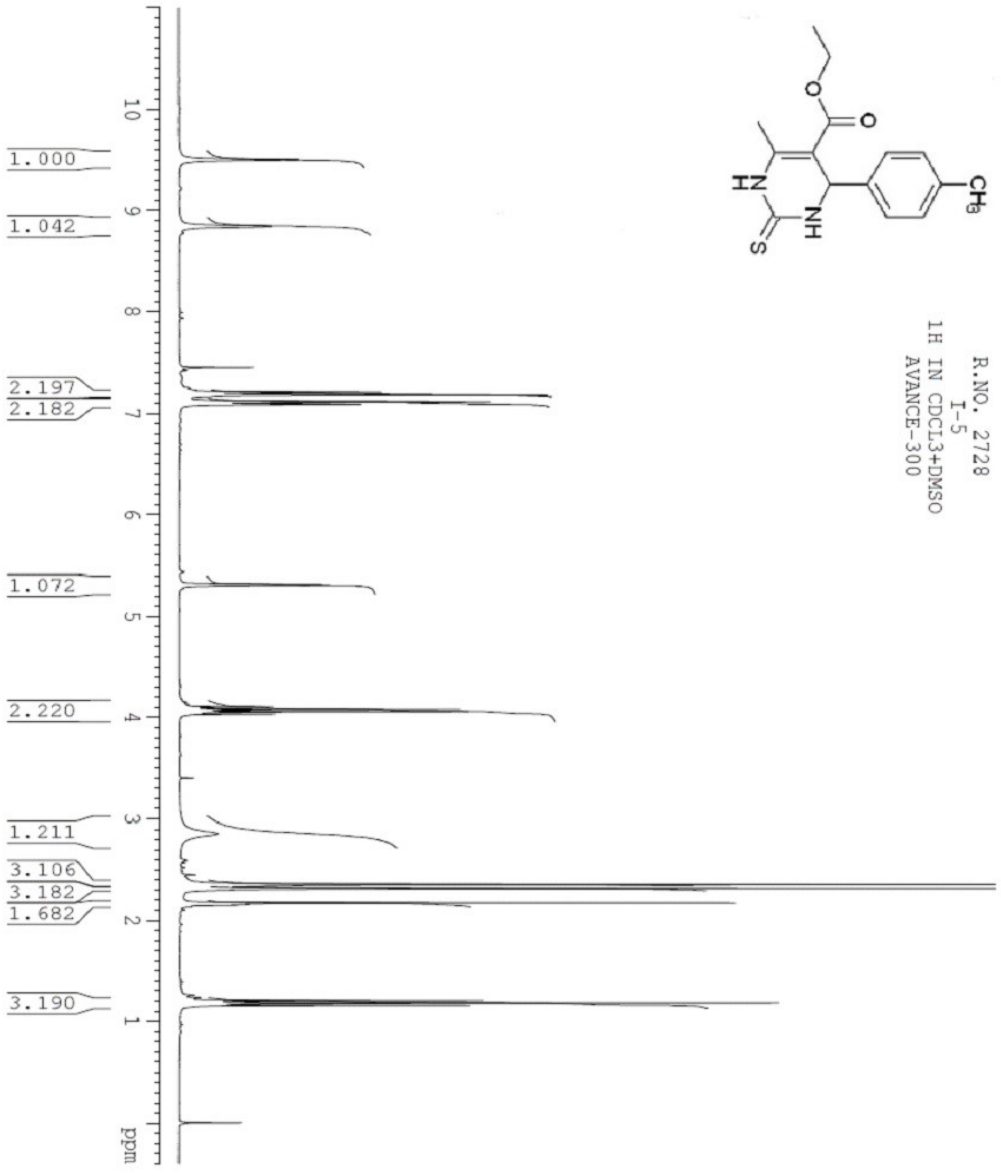

Figure S15: ^{1}H NMR of Compound **1.4**

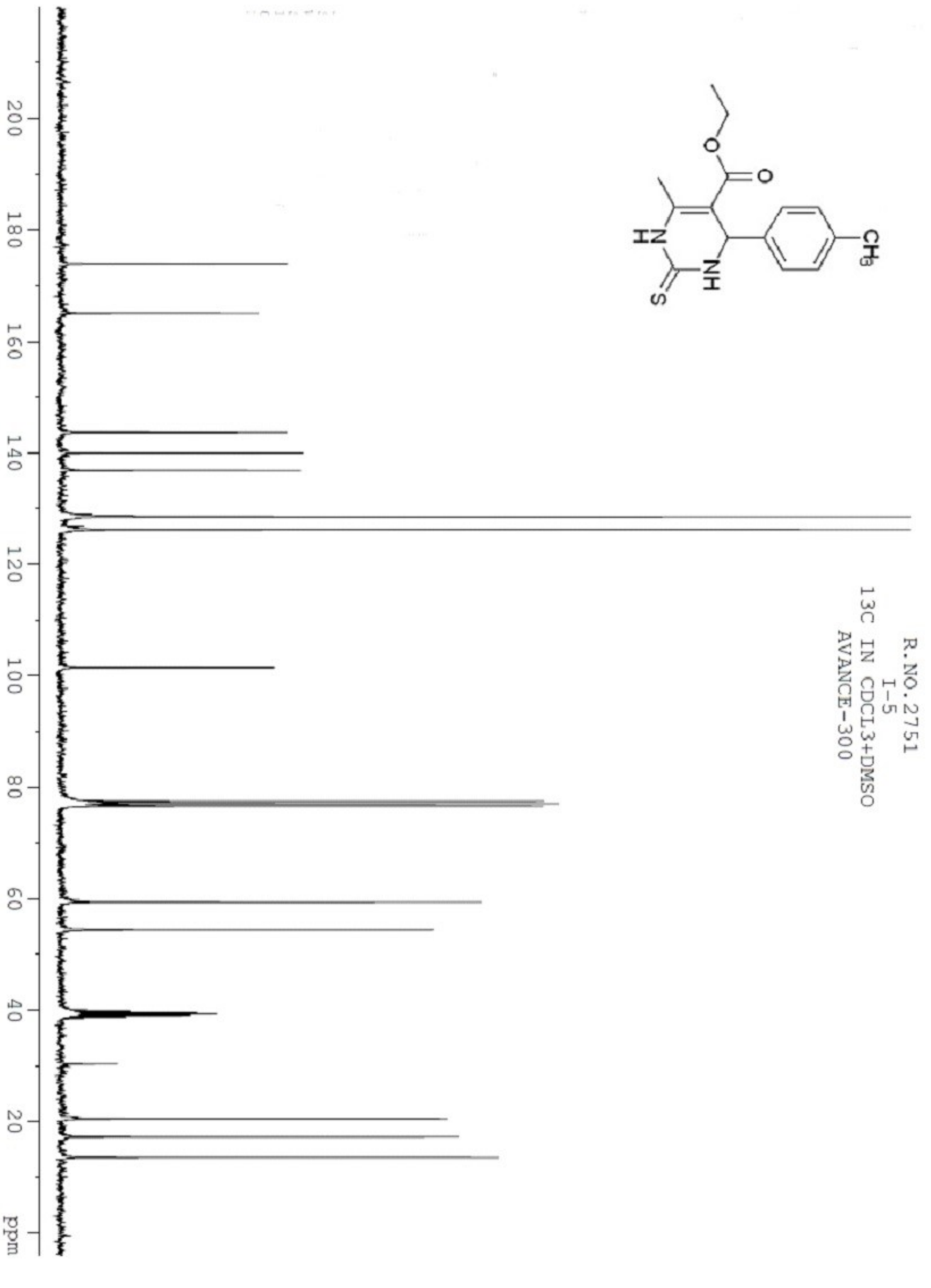

Figure S16: ^{13}C NMR of Compound **1.4**

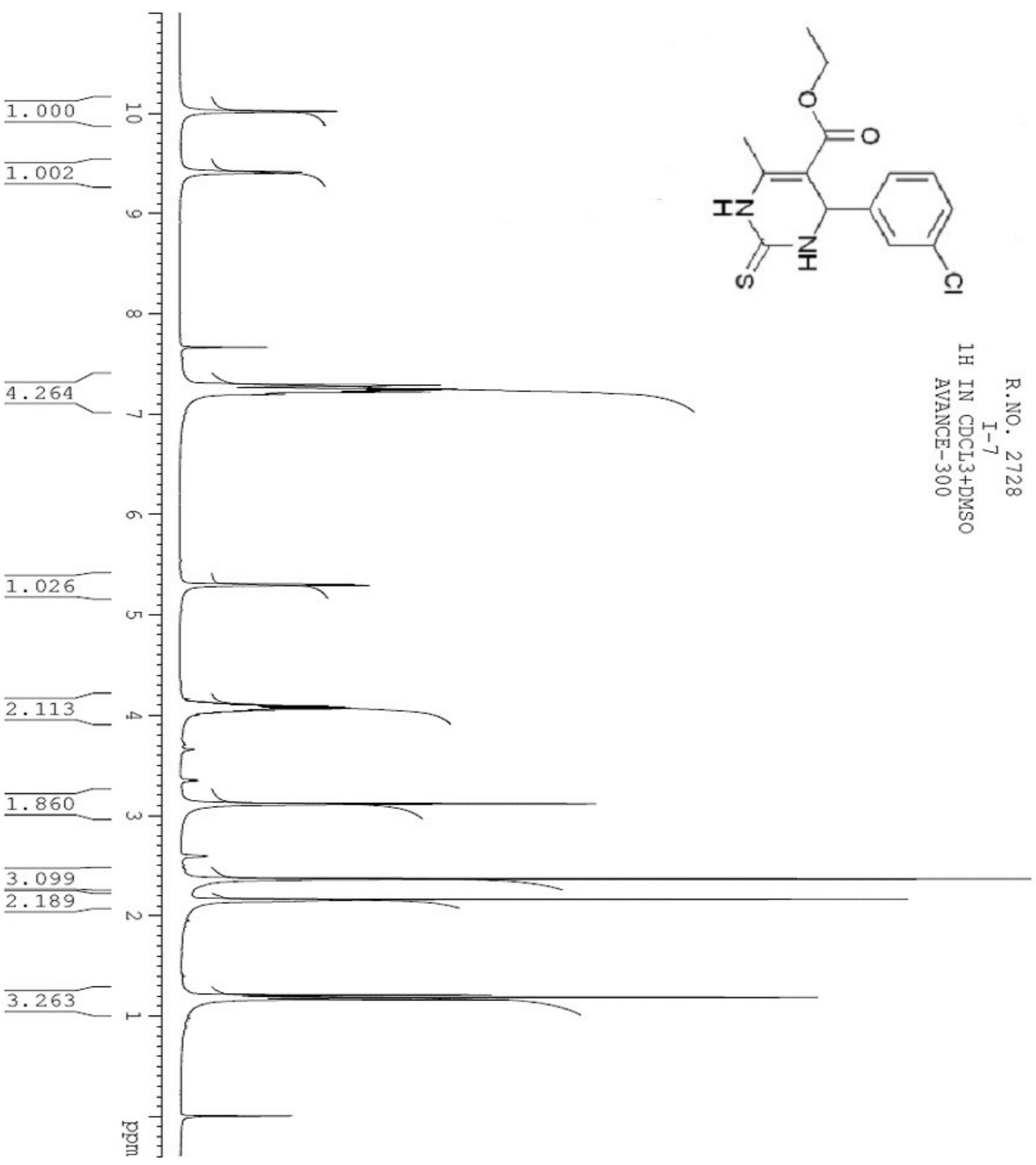

Figure S17: ^{1}H NMR of Compound **1.5**

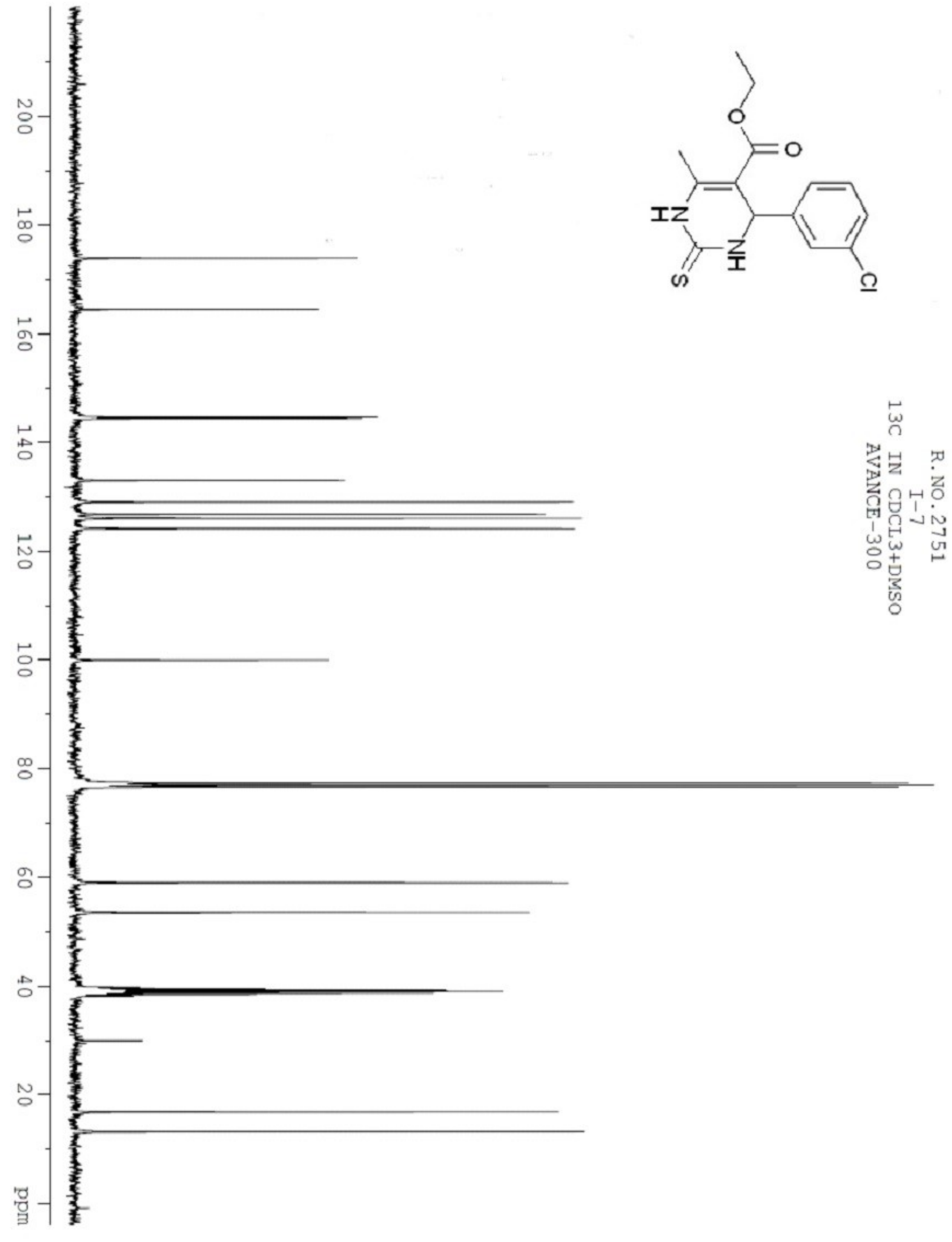

Figure S18: ^{13}C NMR of Compound **1.5**

4.CheckCIF of compound 1.3.

checkCIF/PLATON (basic structural check)

Structure factors have been supplied for datablock(s) VPS_I_4_0ma

THIS REPORT IS FOR GUIDANCE ONLY. IF USED AS PART OF A REVIEW PROCEDURE FOR PUBLICATION, IT SHOULD NOT REPLACE THE EXPERTISE OF AN EXPERIENCED CRYSTALLOGRAPHIC REFEREE.

No syntax errors found.
Please wait while processing ....

CIF dictionary
Interpreting this report

Structure factor report

Datablock: VPS_I_4_0ma

```
Bond precision:        C-C = 0.0037 A              Wavelength=0.71073
Cell:        a=18.2332(17)     b=7.3341(6)      c=25.197(2)
             alpha=90          beta=101.888(4)  gamma=90
Temperature: 296 K
                     Calculated                 Reported
Volume               3297.2(5)                  3297.2(5)
Space group          C 2/c                      C 1 2/c 1
Hall group           -C 2yc                     -C 2yc
Moiety formula       C15 H18 N2 O3 S            C15 H18 N2 O3 S
Sum formula          C15 H18 N2 O3 S            C15 H18 N2 O3 S
Mr                   306.37                     306.39
Dx,g cm-3            1.234                      1.234
Z                    8                          8
Mu (mm-1)            0.207                      0.207
F000                 1296.0                     1297.6
F000'                1297.54
h,k,lmax             24,9,33                    24,9,33
Nref                 4126                       4117
Tmin,Tmax            0.952,0.959
Tmin'                0.952
Correction method= Not given
Data completeness= 0.998          Theta(max)= 28.370
R(reflections)= 0.0636( 3387)       wR2(reflections)= 0.2033( 4117)
S = 1.071                 Npar= 201
```

The following ALERTS were generated. Each ALERT has the format
test-name_ALERT_alert-type_alert-level.
Click on the hyperlinks for more details of the test.

Alert level C

```
PLAT220_ALERT_2_C Non-Solvent  Resd 1  C   Ueq(max)/Ueq(min) Range        3.3 Ratio
PLAT222_ALERT_3_C Non-Solv.  Resd 1  H   Uiso(max)/Uiso(min) Range        4.2 Ratio
PLAT242_ALERT_2_C Low    'MainMol' Ueq as Compared to Neighbors of        C9 Check
PLAT601_ALERT_2_C Structure Contains Solvent Accessible VOIDS of .        91 Ang**3
PLAT906_ALERT_3_C Large K Value in the Analysis of Variance ......     7.815 Check
PLAT906_ALERT_3_C Large K Value in the Analysis of Variance ......     2.153 Check
PLAT911_ALERT_3_C Missing FCF Refl Between Thmin & STh/L=    0.600       2 Report
PLAT918_ALERT_3_C Reflection(s) with I(obs) much Smaller I(calc) .       2 Check
```

Alert level G

```
PLAT072_ALERT_2_G SHELXL First  Parameter in WGHT  Unusually Large       0.11 Report
PLAT083_ALERT_2_G SHELXL Second Parameter in WGHT  Unusually Large       5.15 Why ?
PLAT720_ALERT_4_G Number of Unusual/Non-Standard Labels .........          1 Note
PLAT793_ALERT_4_G Model has Chirality at C6          (Centro SPGR)          R Verify
PLAT883_ALERT_1_G No Info/Value for _atom_sites_solution_primary .     Please Do !
PLAT910_ALERT_3_G Missing # of FCF Reflection(s) Below Theta(Min).          1 Note
PLAT912_ALERT_4_G Missing # of FCF Reflections Above STh/L=  0.600          5 Note
PLAT960_ALERT_3_G Number of Intensities with I  <    - 2*sig(I) ...        12 Check
PLAT978_ALERT_2_G Number C-C Bonds with Positive Residual Density.          8 Info
PLAT983_ALERT_1_G The  S-f"=    0.1244 Deviates from  IT-Value =      0.1234 Check
```

```
   0 ALERT level A = Most likely a serious problem - resolve or explain
   0 ALERT level B = A potentially serious problem, consider carefully
   8 ALERT level C = Check. Ensure it is not caused by an omission or oversight
  10 ALERT level G = General information/check it is not something unexpected

   2 ALERT type 1 CIF construction/syntax error, inconsistent or missing data
   6 ALERT type 2 Indicator that the structure model may be wrong or deficient
   7 ALERT type 3 Indicator that the structure quality may be low
   3 ALERT type 4 Improvement, methodology, query or suggestion
   0 ALERT type 5 Informative message, check
```

It is advisable to attempt to resolve as many as possible of the alerts in all categories. Often the minor alerts point to easily fixed oversights, errors and omissions in your CIF or refinement strategy, so attention to these fine details can be worthwhile. In order to resolve some of the more serious problems it may be necessary to carry out additional measurements or structure refinements. However, the purpose of your study may justify the reported deviations and the more serious of these should normally be commented upon in the discussion or experimental section of a paper or in the "special_details" fields of the CIF. checkCIF was carefully designed to identify outliers and unusual parameters, but every test has its limitations and alerts that are not important in a particular case may appear. Conversely, the absence of alerts does not guarantee there are no aspects of the results needing attention. It is up to the individual to critically assess their own results and, if necessary, seek expert advice.

Publication of your CIF in IUCr journals

A basic structural check has been run on your CIF. These basic checks will be run on all CIFs submitted for publication in IUCr journals (*Acta Crystallographica*, *Journal of Applied Crystallography*, *Journal of Synchrotron Radiation*); however, if you intend to submit to *Acta Crystallographica Section C* or *E* or *IUCrData*, you should make sure that full publication checks are run on the final version of your CIF prior to submission.

Publication of your CIF in other journals

Please refer to the *Notes for Authors* of the relevant journal for any special instructions relating to CIF submission.

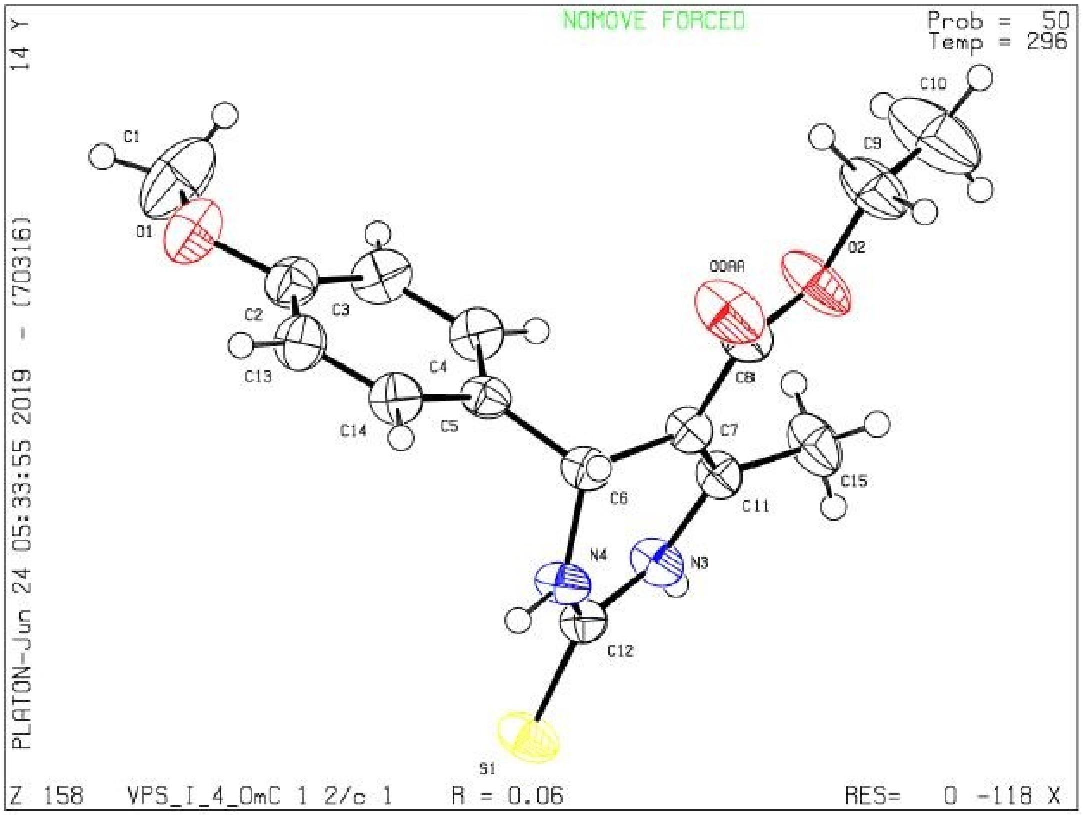
NOMOVE FORCED
Prob = 50
Temp = 296
14 Y
PLATON-Jun 24 05:33:55 2019 - (70316)
Z 158 VPS_I_4_0mC 1 2/c 1 R = 0.06 RES= 0 -118 X
C1
O1
C2
C3
C13
C4
C14
C5
C6
C10
C9
O0AA
O2
C8
C7
C15
C11
N4
N3
C12
S1

5.CheckCIF of compound 1.4.

checkCIF/PLATON (basic structural check)

Structure factors have been supplied for datablock(s) VPS_I_5_0m

THIS REPORT IS FOR GUIDANCE ONLY. IF USED AS PART OF A REVIEW PROCEDURE FOR PUBLICATION, IT SHOULD NOT REPLACE THE EXPERTISE OF AN EXPERIENCED CRYSTALLOGRAPHIC REFEREE.

No syntax errors found. CIF dictionary
Please wait while processing .... Interpreting this report

Structure factor report

Datablock: VPS_I_5_0m

```
Bond precision:       C-C = 0.0025 A              Wavelength=0.71073
Cell:        a=7.3603(10)     b=9.4648(12)     c=12.2076(16)
             alpha=74.216(4)  beta=88.729(4)   gamma=69.819(4)
Temperature: 296 K
```

	Calculated	Reported
Volume	765.70(18)	765.70(18)
Space group	P -1	P -1
Hall group	-P 1	-P 1
Moiety formula	C15 H18 N2 O2 S	C15 H18 N2 O2 S
Sum formula	C15 H18 N2 O2 S	C15 H18 N2 O2 S
Mr	290.37	290.39
Dx,g cm-3	1.260	1.259
Z	2	2
Mu (mm-1)	0.214	0.214
F000	308.0	308.4
F000'	308.36	
h,k,lmax	9,12,15	9,12,15
Nref	3399	3387
Tmin,Tmax	0.954,0.962	
Tmin'	0.954	

```
Correction method= Not given
Data completeness= 0.996          Theta(max)= 27.150
R(reflections)= 0.0423( 2926)        wR2(reflections)= 0.1298( 3387)
S = 1.067              Npar= 192
```

The following ALERTS were generated. Each ALERT has the format
test-name_ALERT_alert-type_alert-level.
Click on the hyperlinks for more details of the test.

Alert level C

```
PLAT193_ALERT_1_C Cell and Diffraction Temperatures Differ by ....          3 Degree
```

Alert level G

```
PLAT154_ALERT_1_G The s.u.'s on the Cell Angles are Equal ..(Note)      0.004 Degree
PLAT199_ALERT_1_G Reported _cell_measurement_temperature ..... (K)        293 Check
PLAT720_ALERT_4_G Number of Unusual/Non-Standard Labels ..........          1 Note
PLAT793_ALERT_4_G Model has Chirality at C6            (Centro SPGR)        R Verify
PLAT883_ALERT_1_G No Info/Value for _atom_sites_solution_primary .     Please Do !
PLAT910_ALERT_3_G Missing # of FCF Reflection(s) Below Theta(Min).          3 Note
```

```
PLAT912 ALERT 4 G Missing # of FCF Reflections Above STh/L=  0.600         10 Note
PLAT960 ALERT 3 G Number of Intensities with I  <   - 2*sig(I) ...          4 Check
PLAT978 ALERT 2 G Number C-C Bonds with Positive Residual Density.          5 Info
PLAT983 ALERT 1 G The  S-f"=    0.1244 Deviates from  IT-Value =       0.1234 Check
```

```
   0 ALERT level A = Most likely a serious problem - resolve or explain
   0 ALERT level B = A potentially serious problem, consider carefully
   1 ALERT level C = Check. Ensure it is not caused by an omission or oversight
  10 ALERT level G = General information/check it is not something unexpected

   5 ALERT type 1 CIF construction/syntax error, inconsistent or missing data
   1 ALERT type 2 Indicator that the structure model may be wrong or deficient
   2 ALERT type 3 Indicator that the structure quality may be low
   3 ALERT type 4 Improvement, methodology, query or suggestion
   0 ALERT type 5 Informative message, check
```

It is advisable to attempt to resolve as many as possible of the alerts in all categories. Often the minor alerts point to easily fixed oversights, errors and omissions in your CIF or refinement strategy, so attention to these fine details can be worthwhile. In order to resolve some of the more serious problems it may be necessary to carry out additional measurements or structure refinements. However, the purpose of your study may justify the reported deviations and the more serious of these should normally be commented upon in the discussion or experimental section of a paper or in the "special_details" fields of the CIF. checkCIF was carefully designed to identify outliers and unusual parameters, but every test has its limitations and alerts that are not important in a particular case may appear. Conversely, the absence of alerts does not guarantee there are no aspects of the results needing attention. It is up to the individual to critically assess their own results and, if necessary, seek expert advice.

Publication of your CIF in IUCr journals

A basic structural check has been run on your CIF. These basic checks will be run on all CIFs submitted for publication in IUCr journals (*Acta Crystallographica*, *Journal of Applied Crystallography*, *Journal of Synchrotron Radiation*); however, if you intend to submit to *Acta Crystallographica Section C* or *E* or *IUCrData*, you should make sure that full publication checks are run on the final version of your CIF prior to submission.

Publication of your CIF in other journals

Please refer to the *Notes for Authors* of the relevant journal for any special instructions relating to CIF submission.

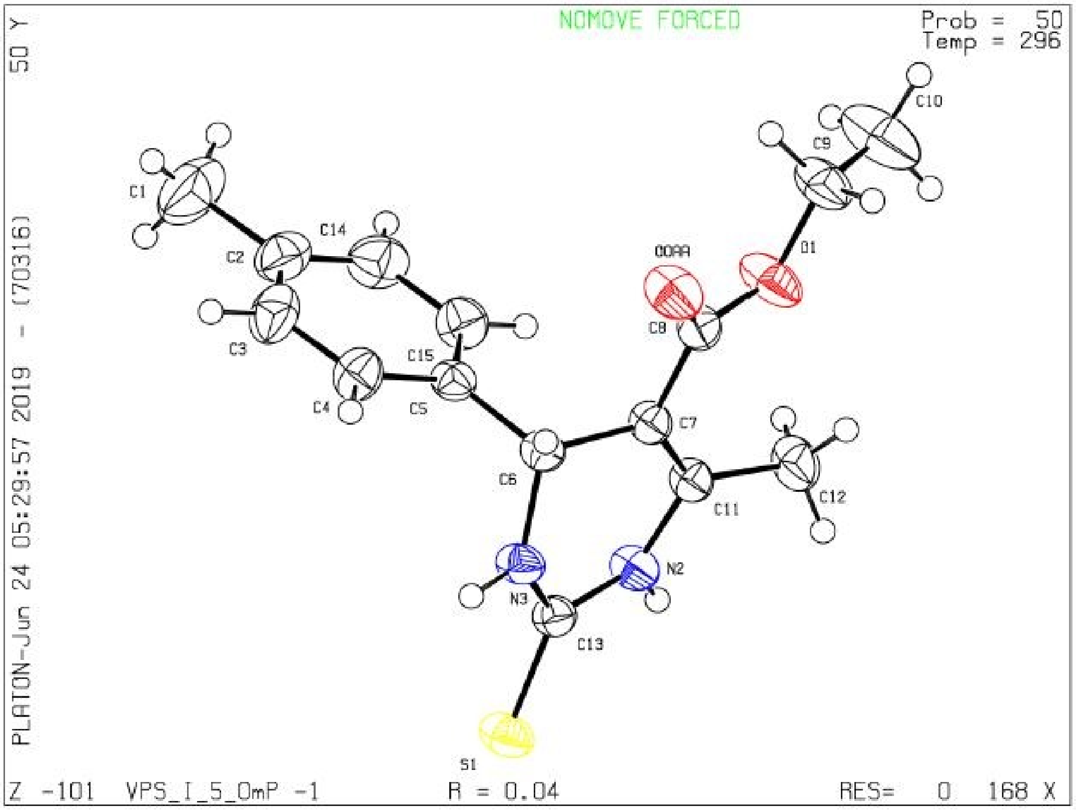
NOMOVE FORCED
Prob = 50
Temp = 296
50 Y
PLATON-Jun 24 05:29:57 2019 - (70316)
C1
C2
C14
C3
C15
C4
C5
C6
C7
C8
O0AA
O1
C9
C10
C11
C12
N2
N3
C13
S1
Z -101 VPS_I_5_OmP -1
R = 0.04
RES= 0 168 X

6.CheckCIF of compound 1.5.

checkCIF/PLATON (basic structural check)

Structure factors have been supplied for datablock(s) VPS_I_7_0m

THIS REPORT IS FOR GUIDANCE ONLY. IF USED AS PART OF A REVIEW PROCEDURE FOR PUBLICATION, IT SHOULD NOT REPLACE THE EXPERTISE OF AN EXPERIENCED CRYSTALLOGRAPHIC REFEREE.

No syntax errors found.
Please wait while processing ....

CIF dictionary
Interpreting this report

Structure factor report

Datablock: VPS_I_7_0m

```
Bond precision:       C-C = 0.0024 A              Wavelength=0.71073
Cell:       a=7.3066(6)      b=10.4657(8)      c=10.6788(9)
            alpha=107.568(3) beta=90.583(3)    gamma=107.829(2)
Temperature: 296 K
```

	Calculated	Reported
Volume	736.37(10)	736.37(10)
Space group	P -1	P -1
Hall group	-P 1	-P 1
Moiety formula	C14 H15 Cl N2 O2 S	C14 H15 Cl N2 O2 S
Sum formula	C14 H15 Cl N2 O2 S	C14 H15 Cl N2 O2 S
Mr	310.79	310.81
Dx,g cm-3	1.402	1.402
Z	2	2
Mu (mm-1)	0.403	0.403
F000	324.0	324.7
F000'	324.66	
h,k,lmax	9,13,13	9,13,13
Nref	3279	3263
Tmin,Tmax	0.901,0.923	
Tmin'	0.901	

```
Correction method= Not given
Data completeness= 0.995          Theta(max)= 27.160
R(reflections)= 0.0384( 2892)       wR2(reflections)= 0.1156( 3263)
S = 1.076              Npar= 191
```

The following ALERTS were generated. Each ALERT has the format
test-name_ALERT_alert-type_alert-level.
Click on the hyperlinks for more details of the test.

Alert level C

```
PLAT220_ALERT_2_C Non-Solvent  Resd 1  C   Ueq(max)/Ueq(min) Range       3.3 Ratio
PLAT222_ALERT_3_C Non-Solv.  Resd 1  H   Uiso(max)/Uiso(min) Range       4.2 Ratio
PLAT242_ALERT_2_C Low    'MainMol' Ueq as Compared to Neighbors of        C8 Check
PLAT910_ALERT_3_C Missing # of FCF Reflection(s) Below Theta(Min).         5 Note
PLAT911_ALERT_3_C Missing FCF Refl Between Thmin & STh/L=    0.600         2 Report
```

Alert level G

```
PLAT720_ALERT_4_G Number of Unusual/Non-Standard Labels ..........         1 Note
PLAT793_ALERT_4_G Model has Chirality at C5          (Centro SPGR)         R Verify
```

```
PLAT883_ALERT_1_G No Info/Value for _atom_sites_solution_primary .     Please Do !
PLAT912_ALERT_4_G Missing # of FCF Reflections Above STh/L=  0.600           9 Note
PLAT960_ALERT_3_G Number of Intensities with I  <    - 2*sig(I) ...          6 Check
PLAT978_ALERT_2_G Number C-C Bonds with Positive Residual Density.           8 Info
PLAT983_ALERT_1_G The Cl-f"=    0.1603 Deviates from  IT-Value =       0.1585 Check
PLAT983_ALERT_1_G The  S-f"=    0.1244 Deviates from  IT-Value =       0.1234 Check
```

```
0 ALERT level A = Most likely a serious problem - resolve or explain
0 ALERT level B = A potentially serious problem, consider carefully
5 ALERT level C = Check. Ensure it is not caused by an omission or oversight
8 ALERT level G = General information/check it is not something unexpected

3 ALERT type 1 CIF construction/syntax error, inconsistent or missing data
3 ALERT type 2 Indicator that the structure model may be wrong or deficient
4 ALERT type 3 Indicator that the structure quality may be low
3 ALERT type 4 Improvement, methodology, query or suggestion
0 ALERT type 5 Informative message, check
```

It is advisable to attempt to resolve as many as possible of the alerts in all categories. Often the minor alerts point to easily fixed oversights, errors and omissions in your CIF or refinement strategy, so attention to these fine details can be worthwhile. In order to resolve some of the more serious problems it may be necessary to carry out additional measurements or structure refinements. However, the purpose of your study may justify the reported deviations and the more serious of these should normally be commented upon in the discussion or experimental section of a paper or in the "special_details" fields of the CIF. checkCIF was carefully designed to identify outliers and unusual parameters, but every test has its limitations and alerts that are not important in a particular case may appear. Conversely, the absence of alerts does not guarantee there are no aspects of the results needing attention. It is up to the individual to critically assess their own results and, if necessary, seek expert advice.

Publication of your CIF in IUCr journals

A basic structural check has been run on your CIF. These basic checks will be run on all CIFs submitted for publication in IUCr journals (*Acta Crystallographica*, *Journal of Applied Crystallography*, *Journal of Synchrotron Radiation*); however, if you intend to submit to *Acta Crystallographica Section C* or *E* or *IUCrData*, you should make sure that full publication checks are run on the final version of your CIF prior to submission.

Publication of your CIF in other journals

Please refer to the *Notes for Authors* of the relevant journal for any special instructions relating to CIF submission.

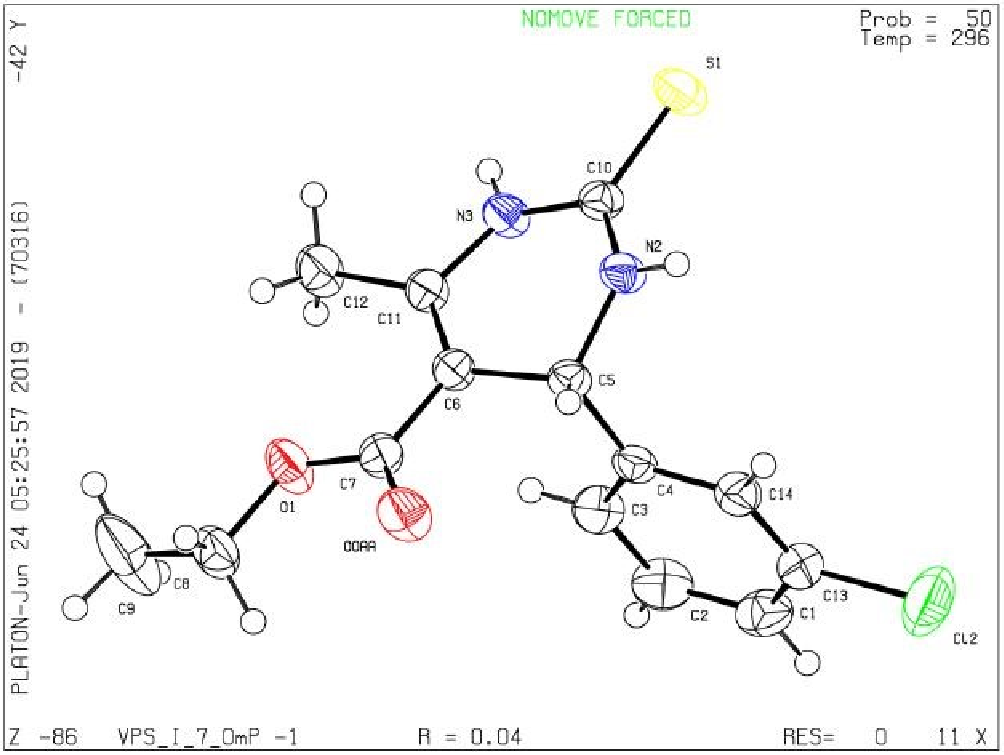
NOMOVE FORCED
Prob = 50
Temp = 296
-42 Y
PLATON-Jun 24 05:25:57 2019 - (70316)
S1
C10
N3
N2
C12
C11
C6
C5
O1
C7
C4
C14
C3
O0AA
C8
C9
C13
C2
C1
CL2
Z -86 VPS_I_7_OmP -1 R = 0.04 RES= 0 11 X
